# Renoprotective effect of Zhenwu decoction against renal fibrosis by regulation of oxidative damage and energy metabolism disorder

**DOI:** 10.1038/s41598-018-32115-9

**Published:** 2018-10-02

**Authors:** Shasha Li, Xue Xiao, Ling Han, Yiming Wang, Guoan Luo

**Affiliations:** 1 Guangdong Provincial Hospital of Chinese Medicine, No. 111 Dade Road, Guangzhou, Guangdong 510120 China; 2 Guangdong Metabolic Diseases Research Center of Integrated Chinese and Western Medicine, Guangdong Pharmaceutical University, Guangzhou, 510006 China; 3 Department of Chemistry, Tsinghua University, No. 30 Shuangqing Road in Haidian Distric, Beijing, 100084 China

## Abstract

Zhenwu decoction (ZWD) is a promising traditional Chinese prescription against renal fibrosis, while its underlying mechanism remains unclear. Rat model of renal fibrosis were established and divided into control group, model group, ZWD treatment group and enalapril maleate treatment group. Metabolic profiles on serum samples from each group were acquired by using ultra performance liquid chromatography coupled with quadrupole time-of-flight high-resolution mass spectrometry. Metabolomics combined with molecular biology were comparatively conducted on samples of various groups. Fifteen potential biomarkers were identified and these biomarkers are mainly phospholipids and fatty acids. The results showed renal fibrosis was associated with oxidative damage and energy metabolism disorder. The results of histopathology, biochemistry and metabolomics demonstrated that ZWD exhibited an efficient renoprotective effect by alleviating oxidative stress, increasing energy metabolism and regulating fibrotic cytokines. This study provided scientific support for the research and development of new drugs from traditional Chinese medicine.

## Introduction

Renal fibrosis is the pathological repair reaction of the kidney under various pathogenic factors such as inflammation and injury. Renal fibrosis characterized a common endpoint of various chronic kidney diseases (CKD) including primary glomerular disease, chronic renal failure, diabetic nephropathy and nephrotic syndrome, which may gradually result in renal dysfunction and even uremia, and seriously endanger the health of human being^1,2^. It is of essentially urgent to effectively prevent the progressive CKD and renal fibrosis.

Nowadays, the rapid development of molecular biology makes it possible to understand the molecular mechanism of renal fibrosis, and accordingly a series of drugs could protect against renal fibrosis^1^. These drugs are mostly pure compounds with clear structure and pharmacological mechanism and they possessed against apoptosis, inflammation and oxidative stress and epithelial-mesenchymal transdifferentiation^3–7^. However, due to the complex mechanism of renal fibrosis and its multiple possible signaling pathways, these single-target drugs may not achieve desired efficacy and safety.

In the recent decades, traditional Chinese medicine (TCM) ever-increasingly attracts worldwide attention for its unique theory, definite efficacy and relatively low toxicity, especially great potentiality in the treatment of CKD and against renal fibrosis^8–12^. Zhenwu decoction (ZWD) is a classical TCM prescription consisting of *Aconiti Lateralis Radix Praeparata*, *Poria cocos*, *Radix Paeoniae Alba*, *Ginger* and *Rhizoma Atractylodis Macrocephalae*. Historically, ZWD was commonly used in clinical for the treatment of kidney disease^13–15^. In our previous study of histopathology and pharmacodynamics, ZWD showed significant treatment effect on renal fibrosis in rats of unilateral ureteral obstruction^16^; however, the mechanism of renoprotective effect of ZWD remains unclear at large.

Metabonomics has been widely applied to biomarker discovery, diagnosis and prognosis and therapeutic evaluation of CKD including chronic glomerulonephritis^17^, diabetic nephropathy^18^ chronic renal failure^19–21^ and patients with hemodialysis^22^. In this study, the approach of metabolomics, combined with serum biochemistry and molecular biological techniques, was applied to discover key biomarkers and signaling pathways associated with ZWD treatment, and is expected to reveal the action mechanisms of biochemistry and molecular biology.

## Materials and Methods

### Instruments and Reagents

Waters UPLC (Waters technologies, USA); AB SCIEX Triple TOF 5600 (AB Sciex, USA); Velocity 14 R high-speed refrigerated centrifuge (Dynamic, Australia); Milli-Q ultrapure water instrument (Millipore, USA); Acetonitrile and formic acid (HPLC grade, Merck, Germany); AB Sciex 5600 Triple TOF correction solution (AB Sciex, USA); Epoch Microplate Spectrophotometer (BioTek, USA); BX63 + DP72 Microscope (Olympus, Japan).

### ZWD Preparation

ZWD was prepared from *Aconiti Lateralis Radix Praeparata* (Habitat: Sichuan; Lot: YDA0L0001), *Poria cocos* (Habitat: Sichuan; Lot: 110805871), *Radix Paeoniae Alba* (Habitat: Anhui; Lot: 110813561), *Rhizoma Atractylodis Macrocephalae* (Habitat: Zhejiang; Lot: 111000441) and *Ginger* (Habitat: Guangzhou, purchased at local market). First, *Aconiti Lateralis Radix Praeparata* (300 g) was decocted with 7 fold water for 1.5 hours; then, *Poria cocos* (300 g), *Rhizoma Atractylodis Macrocephalae* (200 g), *Radix Paeoniae Alba* (300 g) and *Ginger* (300 g) were added after 20 min immersion (Soaking solution discarded) in cold water, and the mixture kept decocting for 30 min. The decoction was filtered and concentrated under decompression to 350 mL. The stock solution was stored in refrigerator under −20 °C.

### Animals and Treatment

Forty specific pathogen-free Sprague Dawley (SD) rats (weight, 180 ± 20 g) were provided by the *Experimental Animal Center of Guangdong Medical* (certificate number, 4407227486). All experiments were performed in accordance with the internationally accepted standard guidelines for the use of animals. This study was conducted in accordance with the Chinese national legislation and local guidelines, and the care and handling of rats were also approved by the *Ethical Committee of Guangdong Provincial Hospital of Chinese Medicine*. The rats were housed under standard environmental conditions (23 ± 2 °C, 55% ± 5% humidity and 12 h/12 h light/dark cycle) and were allowed free access to water as well as standard laboratory rat food.

Ten rats were randomly selected using as control group (CON), and remain thirty rats underwent unilateral ureteral obstruction (UUO) to establish a model of renal fibrosis. UUO rats were randomly divided into three sub-groups after 1 days of routine feeding, i.e. model group (MOD), enalapril maleate (ELM, 10 mg/tablet, purchased from Guangdong Provincial Hospital of Chinese Medicine) treatment group and ZWD treatment group (ZWD). Rats in Group ELM and ZWD were administrated with ELM (10 mg·kg/d) and ZWD (4.55 mL·kg/d), respectively, for 56 days. Rats in CON and MOD group were administrated with equal volume of saline.

Rats were sacrificed after being anesthetized by intraperitoneal injection of pentobarbital (50 mg/kg of body weight) (GBCBIO Technologies, Guangzhou, China) and about 3 mL blood was obtained from abdominal aorta in each rat. Blood samples were drawn into tubes, allowed to stand for 30 min, and were centrifuged to obtain serum. Bilateral kidney tissues were taken and weighed. The surgical kidney was cut transversely, with one half fixed in 10% formalin-PBS solution (Sigma, America) and fixed with paraffin (Leica, Germany) embedded sections. Another half kept at −80 °C centigrade.

Thawed serum (300 μL) was mixed with acetonitrile (1200 μL), then swirled for 2 min and centrifuged at 15,000 rpm for 15 min at 4 °C. The supernatant was transferred to HPLC vial.

### Chromatographic separation

Chromatography was performed on a Waters Acquity UPLC BEH C_18_ column (2.1 mm × 100 mm, 1.7 μm) at 35 °C, with flow rate of 0.4 mL/min. The mobile phase consisted of 0.1% formic acid (Merck, Germany) in water as solvent A and acetonitrile (Merck, Germany) as solvent B. The gradient programs used for separation was as follows: 0–2 min, 98% A, 2% B; 4 min, 75% A, 25% B; 10 min, 50% A, 50% B; 12 min, 35% A, 65%B; 22 min, 15% A, 85% B; 28 min, 2% A, 98%B. Each sample was injected in triplicate with 5 µL injection volume. Each wash cycle consisted of 200 µL of strong wash solvent (80% CH_3_CN-H_2_O, 8:2, v/v) and 600 µL of weak wash solvent (10% CH_3_CN-H_2_O, 1:9, v/v).

### Mass spectrometry condition

MS was performed on AB SCIEX Triple TOF 5600 system equipped with the Duo Spray source (AB Sciex, UK). The ESI probe was used for sample analysis and the APCI probe was used to perform automatic mass calibration through the calibrant delivery system (CDS). Information dependent acquisition (IDA) was used to automatically acquire MS/MS data when an MS signal exceeded a threshold of 3000 cps. MS spectra were acquired in negative ionization mode.

A preliminary experiment was conducted to optimize the experimental conditions. To achieve the desired detection results, the flow rate and column temperature for chromatography, as well as capillary voltage, flow rate of gas and ion source temperature as well as other parameters for the mass spectrometry detector were optimized carefully. As a result, the optimal parameters were fixed as followed.

The flow rate of ion source gas 1, ion source gas 2 and the curtain gas were set as 50 mL/min, 50 mL/min and 35 mL/min, respectively. The ion spray voltage was set to −4500 V at ion source temperature of 500 °C. A TOF-MS survey scan (100–2000 m/z) followed by 6 MS/MS scans (50–1500 m/z) with accumulation time of 0.1 s and 0.08 s respectively. The declustering potential voltage was −100 V. For TOF MS, the collision energy was set to −10 V and set to −35 V with a spread of ±15 V for MS/MS. The ion release delay was 67 and the ion cluster width was 25. Dynamic background substract (DBS) mode was used in this detection.

QC sample is prepared from mixed serum of rats in different groups. The precision and stability of the Ultra performance liquid chromatography-mass spectrometry (UPLC-MS) method was determined by repeated analysis of six injections of the same QC samples, and the repeatability of sample preparation was accessed by preparing six parallel samples using the same protocol. Corrected solution and QC samples analyzed at every 5 injection intervals.

### Data Analysis

The software of Marker View 1.2 version (AB Sciex, USA) was used for acquisition and analysis of mass spectrum data. Data acquisition range was 0.1–28.0 min with 6 peaks was simultaneously detected, and the minimum peak intensity was set as the 10% intensity of the base peak, and the minimum peak width was 50 ppm. The deviation of retention time and m/z are within 0.05 min and 25 ppm respectively.

The processed data was then exported and processed by principle component analysis (PCA) and partial least square-discriminant analysis (PLS-DA) in the software package SIMCA-P 11.5 version (Umetrics AB, Umeå, Sweden). PCA was used first to determine the general interrelation between the groups, and PLS-DA was subsequently performed to maximize the difference in metabolic profiling. The value of Q^2^ and R^2^ were used to estimate the accuracy of the model and a typical 7 round cross validation was used to validate the model against over fitting.

Qualitative analysis of compounds was performed using Peak View software 1.2 version (AB Sciex, USA), by searching the databases of HMDB, PubChem, NIST, MassBank and KEGG according to the information of isotope peak ratio and MS/MS fragments.

Statistical analyses were performed using SPSS software (Version 18.0, USA). Assumptions of normality and homogeneity of variance were first checked. Data were presented as the mean ± standard deviation for continuous variables with a normal distribution, as counts and percentages for categorical variables. The independent samples t-test or one-way ANOVA were used to analyze the differences among groups for continuous measures. Differences with P values less than 0.05 were considered to have statistical differences, and P values less than 0.01 were considered to have significant differences. All probability values were two-sided.

## Result

### Pharmacodynamics evaluation

**Figure 1:**
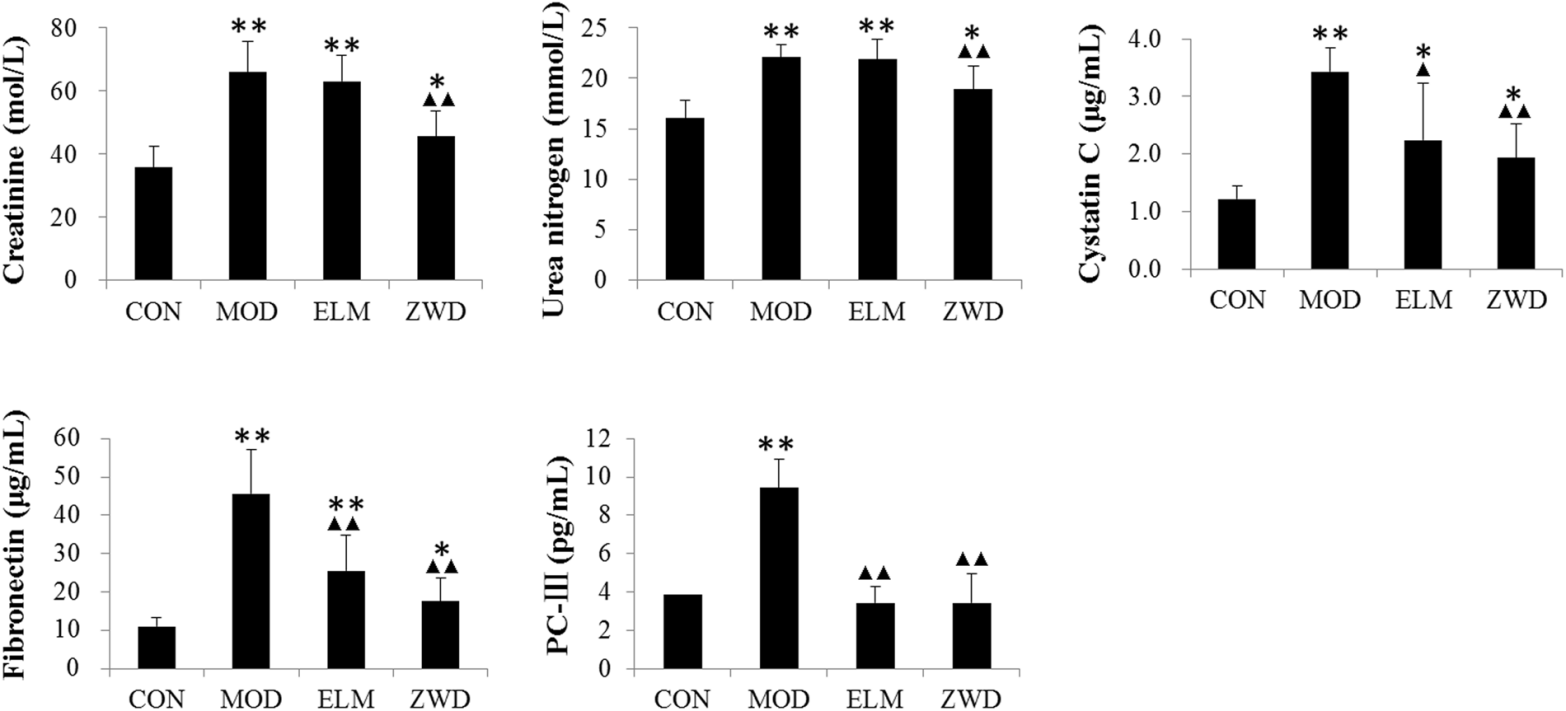
The concentration of blood biochemical indexes in rats of various groups. Data were statistically tested by Dunnett’s *t*-test when the variance in different groups was homogeneous and by the Games-Howell test when the variance was non-homogeneous.^*^P < *0.05*, ^**^P < *0.01* compared with control group; ^▲^*P* < *0.05*, ^▲▲^*P* < *0.01* compared model group. CON, control group; MOD, model group; ELM, enalapril maleate treatment group; ZWD: ZWD treatment group.

UUO rats were clearly observed with depression, loose-hair and inappetence, while these symptoms were much more ameliorated after ZWD administration. Serum creatinine (Medicalsystem, 201711), blood urea nitrogen (Medicalsystem, 201710), fibronectin (Cusabio, 17080403), Type III procollagen (PC-III) (Cusabio, 17060104) and cystatin C (Cusabio, 17081203) were determined. Both serum creatinine and blood urea nitrogen are the clinical biomarkers of renal function. Fibronectin and PC-III are routine markers of fibrosis, and Cystatin C is commonly used to evaluate glomerular filtration function. These five biochemical parameters are determined to evaluate UUO model and reveal the effects of ZWD on renal function in renal fibrosis of rats (shown in Fig. 1).

The five indexes in UUO rats were significantly increased compared with CON group (P < 0.01), while ZWD administration can significantly improve their altered changes. Expect for PC-III, the intervention effect of ZWD better than ELM.

**Figure 2:**
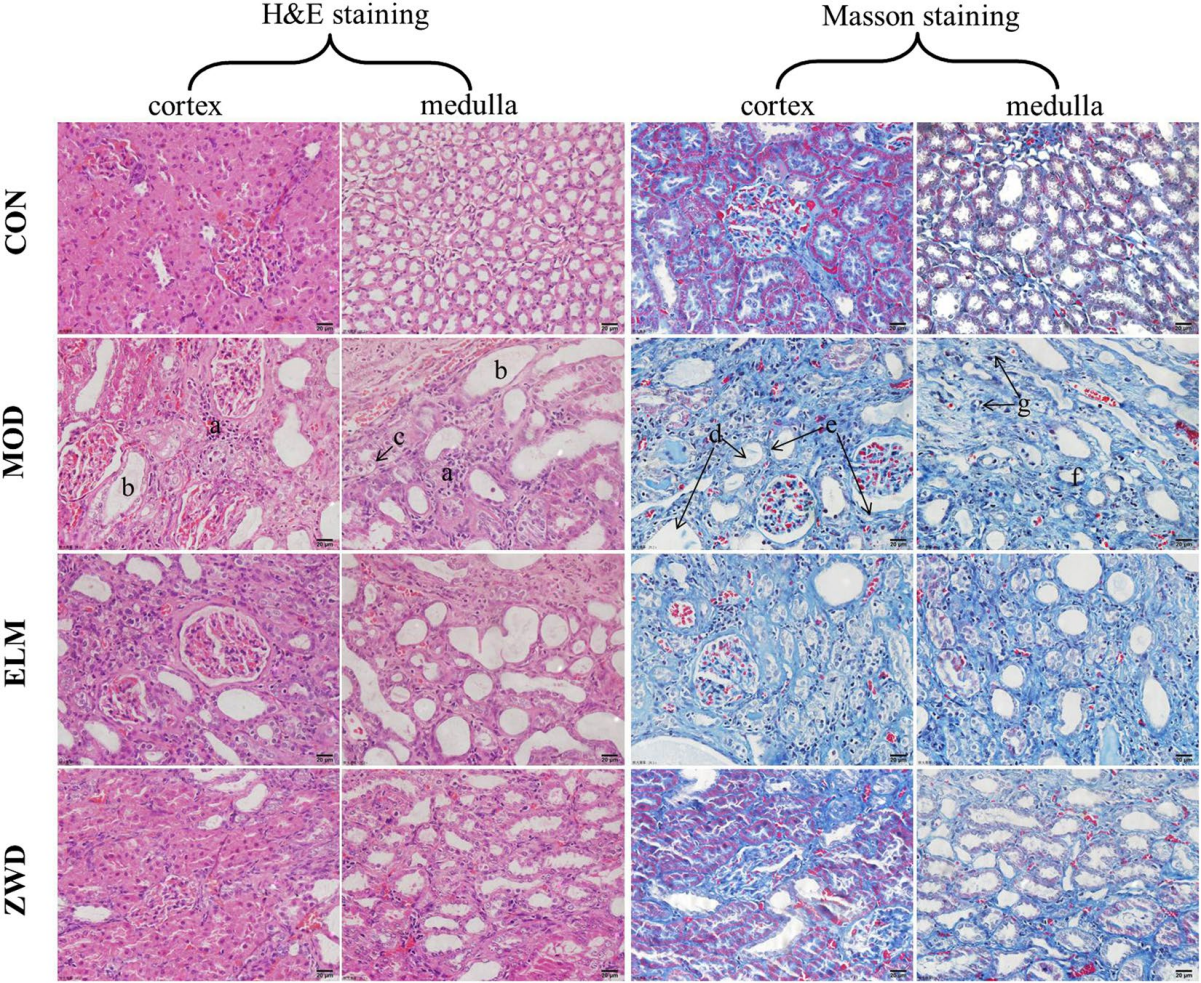
Hematoxylin & Eosin (H&E) staining and Masson staining on kidney tissue of rats in various groups (×400). (**a**) lymphocyte and monocyte cell infiltration; (**b**) protein casts; (**c**) cytoplasmic vacuolation; (**d**) brush border of lumen surface fell off in renal tubular and tubular dilatation; (**e**) fibrotic hyperplasia of glomerular basement membrane and renal interstitium; (**f**) collecting duct; (**g**) medullary loop. CON, control group; MOD, model group; ELM, enalapril maleate group; ZWD: ZWD group.

Hematoxylin & Eosin (H&E) staining and Masson trichrome staining (Fig. 2) showed that UUO rats exhibited lymphocyte and monocyte cell infiltration (a), protein casts (b), cytoplasmic vacuolation (c) and brush border of lumen surface fell off in renal tubular with dilatation (d), fibrotic hyperplasia of glomerular basement membrane and renal interstitium (e), unclear structure of collecting duct (f) and medullary loop (g) in renal medulla, which are similar to diffuse renal fibrosis in clinical. As shown in Fig. 2, ZWD treatment improved the damage in renal cortex and medulla, reduced the degree of inflammatory infiltration and tubulointerstitial fibrosis, as well as improved densely arranged medullary cells with only few fibrous connective tissue hyperplasia. It is worth noting that the content of glomerulus and tubules with normal morphology in kidney of UUO rats was increased obviously after ZWD intervention. To evaluate the degree of damage in kidney, each sample was performed based on 10 randomly selected fields per section, which were examined under x200 magnification. The evaluation criteria and results were shown in Table 1. Results showed that ZWD could effectively alleviate renal fibrosis in rats.

**Table 1:** Evaluation on the degree of damage in kidney.

Type	Criteria				Results^a^			
	0	1	2	3	CON	MOD	ELM	ZWD
Inflammatory Infiltration	none	scatter	focal	diffuse	0	3	1	1
Protein Casts	none	<10%	10–25%	>25%	0	2	2	1
Tubular Dilatation	none	<10%	10–25%	>25%	0	3	2	1
Interstitial fibrosis	none	<25%	25–50%	>50%	0	3	3	2
Total					0	11	7	5

^a^Each sample was performed based on 10 randomly selected fields per section under x200 magnification. CON, control group; MOD, model group; ELM, enalapril maleate group; ZWD: ZWD group.

### Metabolic Profiling

Both positive and negative ionization modes were investigated in this study for metabolic profiling of serum samples. In positive ionization mode, signals of noise and matrix effects are rather high, and signals of low abundance metabolites may be missed; while in negative ionization mode, abundant chromatographic peaks can be detected with acceptable signal-to-noise ratio. Therefore, negative mode is finally adopted in this study.

**Figure 3:**
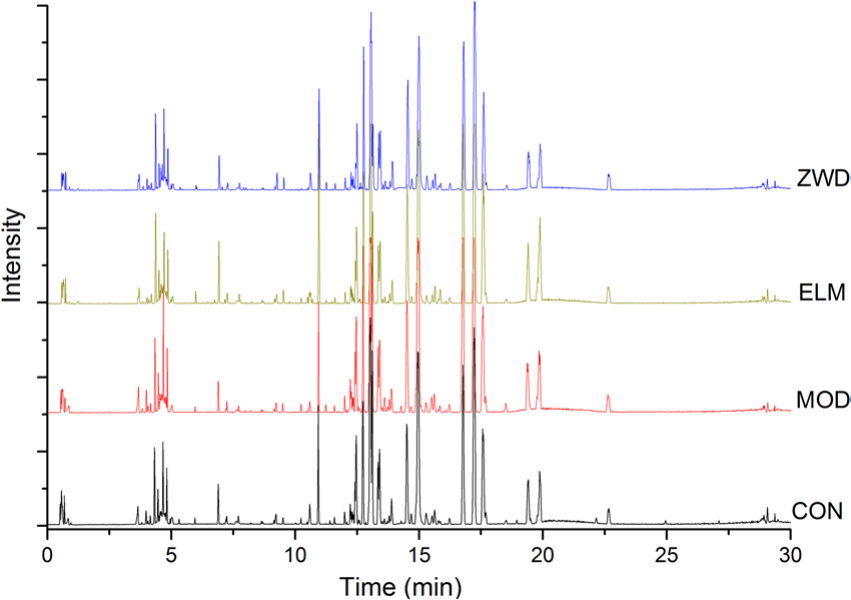
Representative base peak intensity chromatogram of rat serum based on UPLC-QTOF/MS. CON, control group; MOD, model group; ELM, enalapril maleate group; ZWD: ZWD group.

Preliminary experiment suggested that mobile phase containing 0.1% formic acid improved the chromatographic separation of serum samples, especially the acidic metabolites thereof. The flow rate, column temperature, cone voltage, desolvation gas temperature and some other parameters were also optimized, and UPLC-MS conditions were finally determined. Serum samples were conducted UPLC-MS analysis under the optimized conditions to acquire metabolic profiles. The representative profile is shown in Fig. 3.

Ten metabolites were selected (the paired retention time_m/z of these peaks: 5.25_201.0240, 6.89_271.1192, 10.92_768.5371, 12.73_540.3299, 14.51_568.3628, 16.77_327.2342, 17.22_303.2334, 17.59_279.2334, 19.87_281.2494, 22.65_283.2650) in the profile, and the variance of their retention time and peak intensity were investigated for methodological investigation. It was found RSD of retention time and peak intensity was both less than 5%. The metabolic profiling method is thus reliable and reproducible, and is qualified for the subsequent metabolomic analysis.

### Pattern Recognition

**Figure 4:**
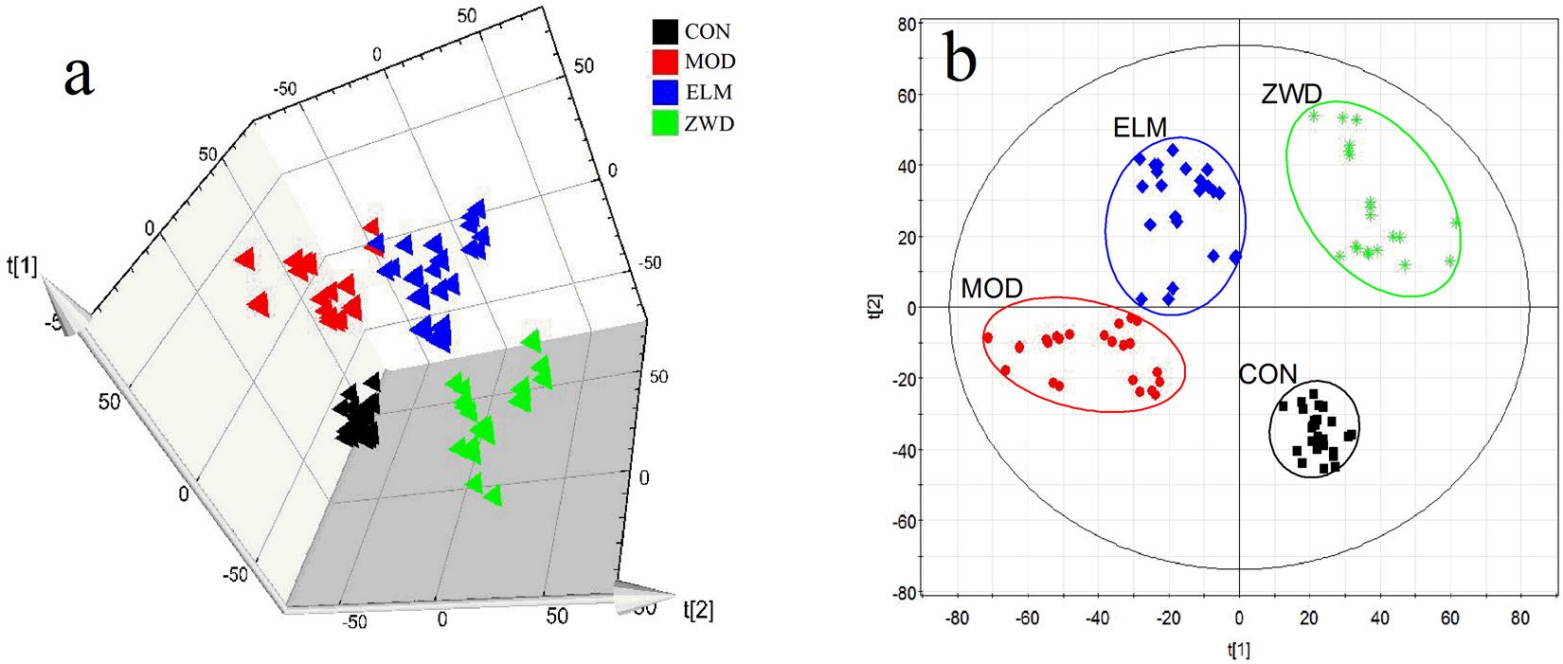
3D Score plots of rat serum data conducted by PCA analysis (**a**) and 2D Score plots of rat serum data conducted by PLS-DA analysis. (**b**) In PLS-DA score plots, the R^2^X(cum), R^2^Y(cum) and Q^2^Y(cum) values is 0.570, 0.968 and 0.938 respectively. CON, control group; MOD, model group; ELM, enalapril maleate group; ZWD: ZWD group.

Pareto scaling was used to reduce the significance of the intensity so that variables have equal importance regardless of the magnitude in this study. Principal component analysis (PCA) was first conducted as unsupervised methods on the normalized UPLC-MS data to give the comprehensive view of the clustering trend (Fig. 4a). In PCA 3D score plot, the CON and MOD group were separated clearly, indicating that the UUO rats have significantly altered endogenous metabolism in negative ion mode. This indicates that metabolic fingerprints could reflect the alternation between UUO and normal rats. In addition, clear classification of ZWD group was also observed, which were away from MOD group, indicating that the treatment of ZWD could affect metabolic pattern of UUO rats. Furthermore, it also indicated that ZWD treatment on UUO rats is better renoprotective effect than ELM, presented as scattered points of ZWD group were relatively more close to CON group than points of ELM group as shown in Fig. 4a.

The results of PCA score plot, clinical biochemistry and histopathology demonstrated that the UUO model was successfully established, and the renal fibrosis could be prevented by treatment ZWD, exhibiting a tendency recovering to CON group after taking ZWD, and ZWD shown better efficacy than ELM against renal fibrosis.

To maximize the difference in metabolic profiling and to find the metabolites with a significant concentration change, partial least squares-discriminate analysis (PLS-DA) was conducted subsequently as shown in Fig. 4b. The R^2^Y and Q^2^Y values calculated by using SIMCA-P package were 0.968 and 0.938, respectively. Meaning 96.8% of data fit the model and 93.8% of data could be predicted by this model. Both the Q^2^Y and R^2^Y close to 1 indicate an excellent model which is good to fitness and prediction.

### Potential Biomarkers

**Figure 5:**
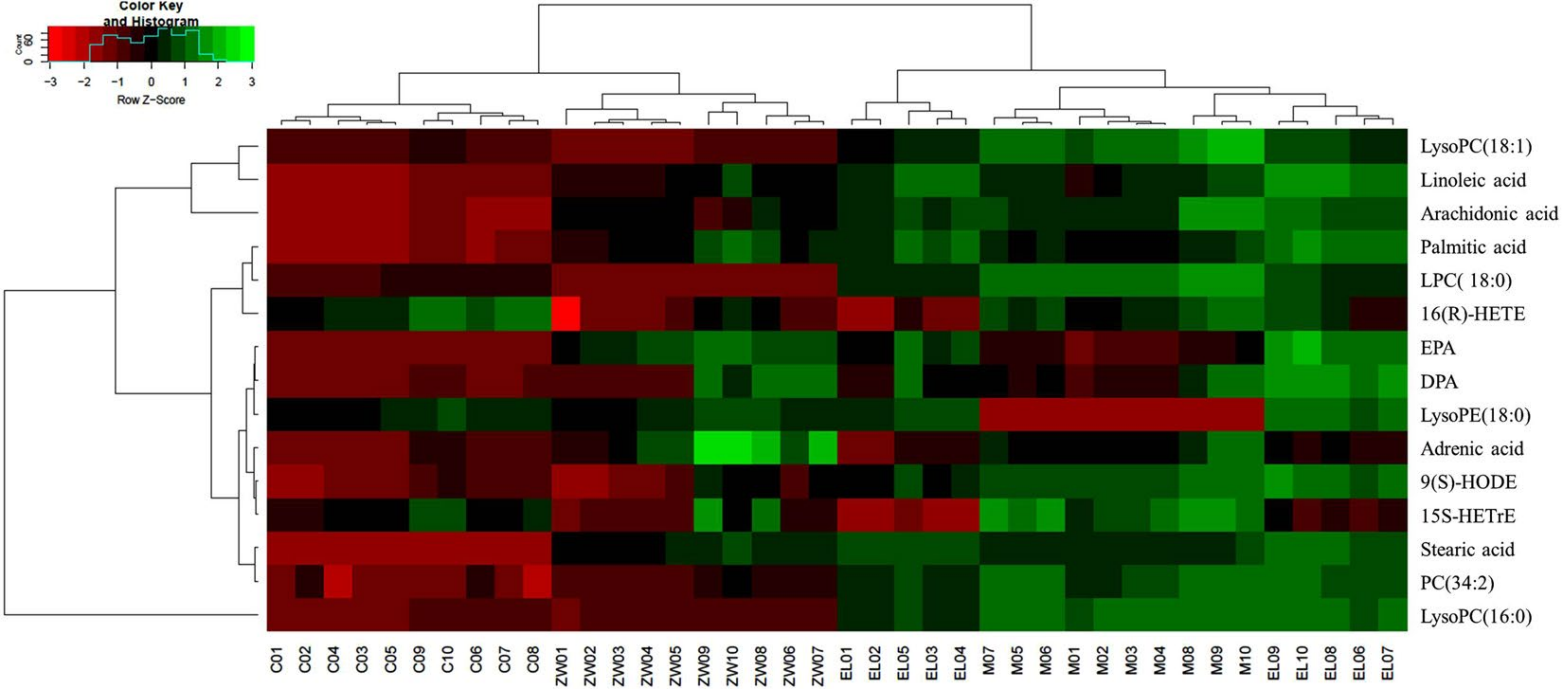
The heat map of 15 potential biomarkers between groups. The color of each section is proportional to the significance of change of metabolites (red, upregulated; green, downregulated) as the numbers listed in Table 2. Rows: metabolites; Columns: samples (C: control group; M: model group; EL: enalapril maleate group; ZW: ZWD group). 16(R)-HETE, 16R-Hydroxy-5Z,8Z,11Z,14Z-eicosatetraenoic acid; EPA, Eicosapentaenoic acid; DPA, Docosapentaenoic acid; 9(S)-HODE, 9S-Hydroxy-10E,12Z-octadecadienoic acid; 15S-HETrE, 15(S)-Hydroxyeicosatrienoic acid.

Metabolites that significantly contributed to the clustering and discrimination were identified, with VIP value (variable importance in projection) of PLS-DA over 1.5 and p-value of t-test between groups less than 0.05. Finally, 15 potential biomarkers were identified and listed in Table 2. The relative levels of biomarkers were also analyzed and the heat map was constructed (Fig. 5).

**Table 2:** Identification of significantly different potential endogenous metabolites in the sera of rats^a^.

No.	Identified potential biomarker	Related pathway	Concentration (Intensity/1000)^b^				F^c^	Sig.^c^	*p* value^d^		
			CON	MOD	ELM	ZWD			CON VS MOD	CON VS ZWD	MOD VS ZWD
1	16(R)-HETE	Arachidonic acid metabolism	15.07 ± 0.99	14.99 ± 0.70	12.89 ± 1.68	12.29 ± 1.73	11.22	2.42E-5	9.01E-1	3.3E-3	3.76E-3
2	LysoPC(18:1)	Glycerophospholipid metabolism	25.03 ± 0.58	31.09 ± 1.27	28.31 ± 0.72	24.35 ± 0.56	140.03	6.65E-20	3.67E-8	8.22E-2	1.25E-8
3	LysoPC(16:0)	Glycerophospholipid metabolism	65.06 ± 1.13	86.32 ± 1.29	82.65 ± 3.99	66.27 ± 1.08	240.93	7.52E-24	3.33E-16	1.33E-1	4.32E-21
4	LysoPC(18:0)	Glycerophospholipid metabolism	9.97 ± 0.15	11.34 ± 0.17	10.37 ± 0.19	8.66 ± 0.10	565.73	2.51E-30	5.77E-15	8.71E-9	2.88E-15
5	LysoPE(18:0)	Glycerophospholipid metabolism	3.52 ± 0.082	2.85 ± 0.046	3.76 ± 0.11	3.59 ± 0.11	189.56	4.19E-22	8.58E-12	6.19E-1	1.72E-9
6	Palmitic acid	Fatty Acid Biosynthesis	7.70 ± 0.40	10.68 ± 0.44	12.10 ± 0.73	10.64 ± 1.04	69.00	5.35E-15	3.62E-11	1.80E-5	1.00
7	Linoleic acid	Linoleic acid metabolism	16.89 ± 0.87	24.01 ± 1.14	27.34 ± 1.65	22.46 ± 1.06	128.75	2.66E-19	1.08E-10	1.65E-9	3.22E-2
8	Stearic acid	Fatty Acid Biosynthesis	1.25 ± 0.09	6.08 ± 0.12	6.58 ± 0.24	5.99 ± 0.28	254.18	2.83E-24	6.65E-16	1.35E-8	5.44E-1
9	EPA	Fatty Acid Biosynthesis	1.91 ± 0.07	2.54 ± 0.25	3.96 ± 0.67	3.72 ± 0.32	61.29	3.21E-14	7.23E-5	5.35E-8	2.96E-7
10	DPA	Fatty Acid Biosynthesis	1.93 ± 0.06	2.48 ± 0.35	2.90 ± 0.45	2.56 ± 0.51	11.004	2.84E-5	4.19E-3	2.00E-2	9.99E-1
11	Adrenic acid	Fatty Acid Biosynthesis	1.08 ± 0.06	1.29 ± 0.09	1.16 ± 0.07	1.42 ± 0.18	17.24	4.17E-7	6.77E-5	1.22E-3	3.38E-1
12	PC(34:2)	Glycerophospholipid metabolism	3.74 ± 0.70	6.67 ± 0.46	6.29 ± 0.41	4.68 ± 0.37	75.50	1.34E-15	4.76E-8	1.17E-2	3.48E-8
13	9(S)-HODE	Linoleic acid metabolism	0.53 ± 0.04	0.78 ± 0.02	0.75 ± 0.08	0.85 ± 0.10	36.89	4.47E-11	2.53E-10	5.75E-1	4.06E-4
14	15S-HETrE	γ-Linolenic acid metabolism	0.28 ± 0.02	0.33 ± 0.02	0.22 ± 0.05	0.27 ± 0.05	21.058	4.74E-8	2.17E-4	9.09E-1	5.46E-3
15	Arachidonic acid	Arachidonic acid metabolism	33.09 ± 0.83	46.87 ± 3.10	16.98 ± 2.14	41.74 ± 1.73	95.27	3.48E-17	3.95E-7	1.45E-8	2.96E-3

^a^Metabolites were confirmed by *t*_R_ and *m*/*z* with authentic chemicals. ^b^The concentration of potential biomarkers in sera of rats are presented as the mean ± standard deviation. ^c^The value of F and Sig. was obtained from a one-way ANOVA. The F value is the ratio of the variance between the groups and the variance in the group. F value greater than 1 and Sig. less than 0.05 means the differences between groups were statistically significant. ^d^p values were calculated from the Dunnett’s t-test in multivariate statistical analysis. 16(R)-HETE, 16R-Hydroxy-5Z,8Z,11Z,14Z-eicosatetraenoic acid; EPA, Eicosapentaenoic acid; DPA, Docosapentaenoic acid; 9(S)-HODE, 9S-Hydroxy-10E,12Z-octadecadienoic acid; 15S-HETrE, 15(S)-Hydroxyeicosatrienoic acid. CON, control group; MOD, model group; ELM, enalapril maleate group; ZWD: ZWD group.

The biomarkers mainly included unsaturated fatty acids, saturated fatty acids and phospholipids, which suggested that renal fibrosis was associated with the abnormal metabolism of arachidonic acid, linoleic acid, glycerophospholipid and fatty acid synthesis pathway.

## Discussion

Lipids are mainly the potential biomarkers in the current study. One category is mainly the lysophospholipids, which may accelerate the development of renal fibrosis^23,24^, such as LysoPC and LysoPE. Another one is fatty acids that play an active therapeutic role in kidney disease, such as EPA, linoleic acid, 15S-HETrE and 9(S)-HODE^25,26^.

The lysophospholipids can influence many of the biological processes involved in wound healing by virtue of their ability to mediate many basic cellular functions. Lysophospholipids were reported to have profibrotic effects by promoting fibroblast migration and persistence to apoptosis, activating latent transforming growth factor β (TGF-β) to increase the production and secretion of platelet-derived growth factor-B (PDGF-B) and connective tissue growth factor (CTGF)^27–29^. There were evidences that lysoPCs are involved in energy metabolism and oxidative stress. Among them, the impact on energy metabolism is mainly presented as inducing insulin resistance via JNK activation and inhibiting Na^+^-K^+^-ATPase competitively^30,31^. And the impact on oxidative stress is mainly presented as decrease of SOD and glutathione peroxidase in addition to rapid increase of reactive oxygen species (ROS) levels. LysoPC is a major lipid constituent of oxidized LDL and plays an important role in oxidized LDL-induced endothelial dysfunction^32–34^. The results showed that the levels of LysoPCs were significantly increased in the UUO rats, while were restored to normal level after the treatment of ZWD. It indicated that ZWD could regulate energy metabolism, alleviate oxidative stress and inhibit the fibrosis in rats by reducing the levels of serum lysophospholipids.

EPA, Linoleic acid, 15S-HETrE and 9(S)-HODE belong to fatty acids with an active therapeutic role in renal fibrosis^35^. Among, EPA is recognized to be a key factor to improve the ratio of urinary albumin/creatinine, and to reduce the glomerular mesangial cell matrix accumulation and tubulointerstitial fibrosis^36^. Linoleic acid is an essential component of cell membranes and biological enzymes with various functions, such as enhancing immune, inhibiting inflammatory and reducing serum lipids^35,37^. Besides, linoleic acid could increase the level of cyclooxygenase-2 (COX-2) protein expression, which stimulated the synthesis of prostaglandin E2 (PGE2). The increase in PGE2 production subsequently stimulated peroxisome proliferator-activated receptor expression (PPAR α and δ) to promote glucose production^37^. 15S-HETrE^38^ can promote the expression of PPARγ, and 9(S)-HODE^39^ is an endogenous PPARγ agonist. All the PPAR nuclear receptor subfamily members, PPARα, PPARβ/δ and PPARγ, are critical in regulation of insulin sensitivity, adipogenesis, lipid metabolism, inflammation, and blood pressure. More, PPARγ exerts anti-fibrosis actions by inhibiting the expression of TGF-β1 and the proliferation of mesangial cells, as well as reducing the accumulation of extracellular matrix fibronectin and type I collagen^40^. As listed in Table 2, the levels of above four potential biomarkers increased in model group, and ZWD did not affect this change. As reported, renal fibrosis is one type of wound healing process without self-limited under various pathogenic factors in kidney disease^41^. The increased content of these four metabolites in UUO rats may be related to this self-healing mechanism, and ZWD did not show regulation effect on them.

In addition to the above metabolites, other potential biomarkers associated with Arachidonic acid metabolism (16(R)-HETE and Arachidonic acid) and biosynthesis of unsaturated fatty acids (DPA, Adrenic acid, Palmitic acid and Stearic acid) were also identified in this work. Both Palmitic acid and Stearic acid are involved in the β -oxidation of fatty acids and play an important role in the energy and fatty acids metabolism^42^. And Palmitic acid had been reported to induce apoptosis of renal tubular epithelial cells and to increased cytosolic phospholipase A2 (cPLA2) and cyclooxygenase 2 (COX-2) that related to the level or action of TGF-β1^43–46^.

The result showed (Table 2) that ZWD had little effect on the content of fatty acids related to self-healing mechanism, and its effect on endogenous metabolites in UUO rats was mainly reflected in the regulation of the content of phospholipid components. Based on the present study, we concluded that the renoprotective effect of ZWD against renal fibrosis in rats is mainly involved in energy metabolism, oxidative stress and fibrosis cytokines regulation. To verify this conclusion, some indicators related to energy metabolism, oxidative stress and fibrosis cytokines were detected subsequently.

### Verification

**Figure 6:**
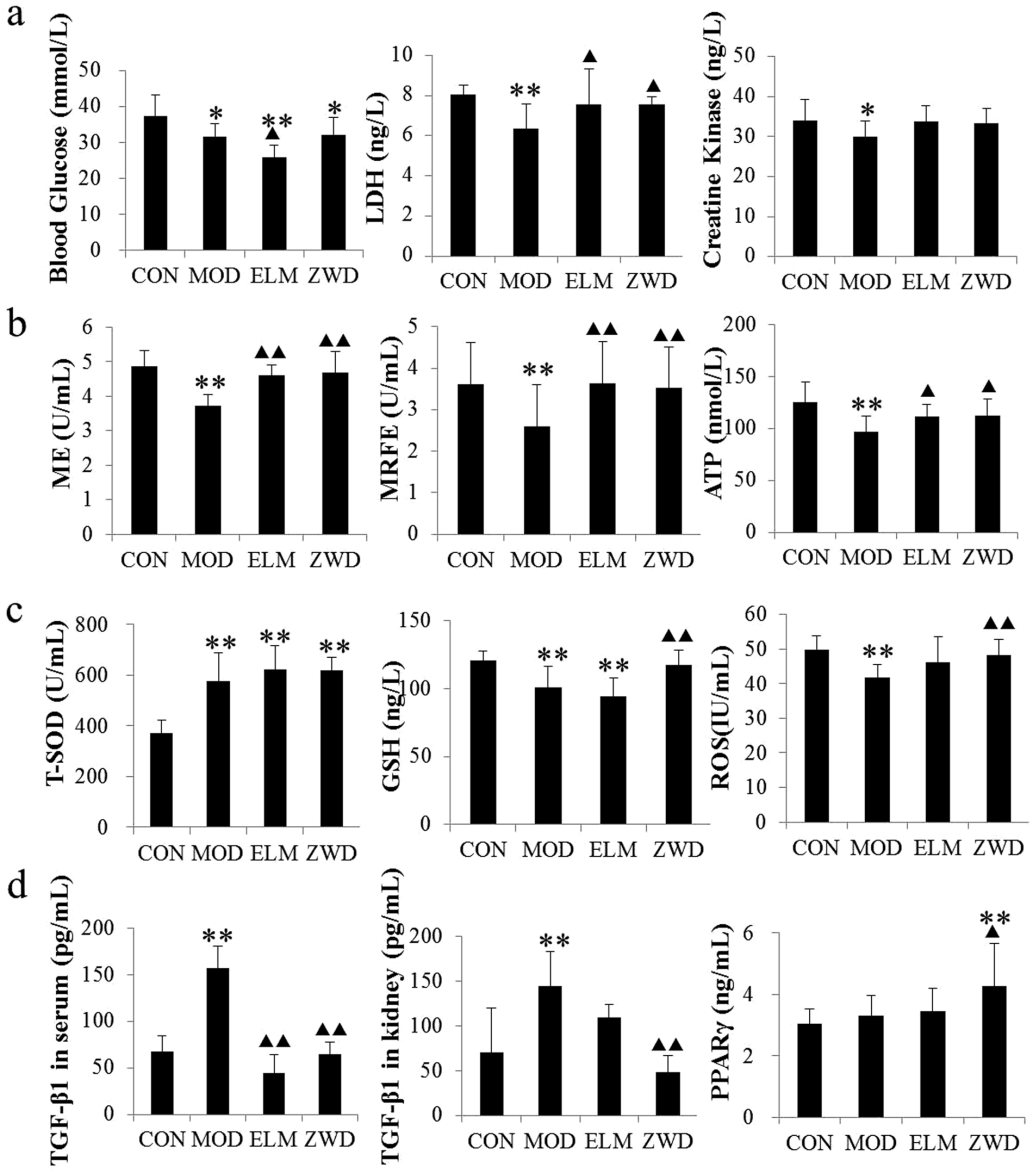
The levels of indicators related to energy metabolism (**a**,**b**), oxidative stress (**c**) and fibrotic cytokines (**d**) in rats presented as the mean ± standard deviation. LSD method is used for multiple comparisons if the variance is homogeneous; otherwise the Games-Howell method is used. ^*^P < *0.05*, ^**^P < *0.01* compared with control group; ^▲^*P* < *0.05*, ^▲▲^*P* < *0.01* compared model group. CON, control group; MOD, model group; ELM, enalapril maleate group; ZWD: ZWD group.

The selection of detective indicators was based on reports of pathways involved in potential biomarkers that were identified in the present study. Glucose (Mlbio, 201709), lactate dehydrogenase (LDH) (Mlbio, 201710), ATP (Mlbio, 201804), mitochondrial enzyme (ME) (Mlbio, 201805), mitochondrial respiratory function enzyme (MRFE) (Mlbio, 201804) and creatine kinase (CK) (Mlbio, 201712) were selected to investigate effect of ZWD on energy metabolism in UUO rats. T-SOD (Mlbio, 201711), Glutathione (GSH) (Mlbio, 201803) and reactive oxygen species (ROS) (mlbio, 201804) were selected as representative indicators involved in oxidative stress. In addition, TGF-β1 (Cusabio, 17120504) and PPARγ (Cusabio, 17100306) were detected as renal fibrosis cytokines in this study. The levels of blood glucose and LDH in serum were detected, and level of CK in ligated kidney was detected as CK is mainly located in tissues. Subsequently, the levels of ATP, ME and MRFE in kidney were also detected to evaluate mitochondrial function which was shown to be associated with the progression of fibrosis^47^. It revealed that decreased levels of blood glucose, LDH and CK were determined in UUO rats (P < 0.05) (shown in Fig. 6a), as well as decreased levels of ATP, ME and MRFE (P < 0.01) (shown in Fig. 6b), suggesting abnormal energy metabolism in rats of renal fibrosis. And the levels increased after ZWD treatment, mainly manifested in the increase of LDH and indicators related to mitochondrial function, indicating an overall regulative effect of ZWD on energy metabolism in renal fibrosis rats, especially on aerobic respiration.

Oxidative stress, resulting in generation of reactive oxygen species (ROS), plays a critical role in the initiation and progression of fibrotic diseases^48^. In the present study, T-SOD activity and content of GSH in serum and ROS in kidney were detected. Among them, T-SOD is an important antioxidant enzyme in organisms, which are widely distributed in various organisms to eliminate free radicals and repair damaged cells^49^. The vitality of T-SOD reflects the ability of the organism to scavenge oxygen free radicals, and it is an intuitive index often used to observe the aging, injury and even death of organisms and cell^50^. GSH system is the most important endogenous defense system against oxidative stress in body as several antioxidant systems depend on glutathione^51^. GSH plays a key role in protecting cells against electrophiles and free radicals. ROS could directly incites damage to biologically important macromolecules and leads to generation of the so-called advanced oxidation protein products (AOPPs) and advanced glycation end products, which are not only markers of oxidative stress but also cause renal injury^52^. In this study, T-SOD activity and level of glutathione (GSH) in serum, as well as content of ROS in kidney were quantitatively analyzed to investigate the effect of ZWD on oxidative stress in UUO rats. Compared with the control rats, T-SOD activity increased and the level of GSH and ROS decreased significantly in model group, suggesting abnormal oxidative stress in rats of renal fibrosis. After ZWD treatment, the increase T-SOD activity maintained a high level and content of GSH and ROS returned to normal as shown in Fig. 6c, indicating an alleviative effect of ZWD on oxidative stress in UUO rats. As oxidative stress and abnormal energy metabolism would lead to a serious of abnormal biochemical processes, such as apoptosis^53^, inflammation^54–56^ and fibrotic cytokine accumulation^57–59^. Fibrosis cytokines including TGF-β1 and PPARγ were also performed by ELISA in serum of rats and TGF-β1 levels in kidney were also presented to ensure the indication (Fig. 6d).

TGF-β1 is one of the most important potent fibrotic factors with functions in cell growth, differentiation and apoptosis. TGF-β1 can be synthesized and released in inflammatory cell in injury kidney, which may induce renal tubular epithelial cells transdifferented into myofibroblasts and promote the fibrosis process^60–62^. PPARγ is a nuclear receptor with multiple biological effects, including inhibiting the expression of TGF-β1 and NF-κB, restraining the proliferation of mesangial cells, decreasing extracellular matrix fibronectin and type I collagen accumulation^63–65^. PPARγ also has effects of anti-inflammatory, anti-fibrosis, regulating lipid metabolism and other biological functions. Figure 6c indicated that ZWD down regulated the level of TGF-β1 expression, while up regulated the expression of PPARγ, which should be responsible for its renoprotective effect.

## Conclusion

This study investigates the renoprotective effects of ZWD against renal fibrosis in UUO rats by using UPLC-MS-based metabolomics. The results of biochemistry, histopathology and metabolomics demonstrated that ZWD treatment exhibited an efficient renoprotective effect by increasing energy metabolism, alleviating oxidative stress and regulating fibrotic cytokines.

## Electronic supplementary material

Dataset 1

## Data Availability

All data generated during this study are included in this published article and the raw data are available from the corresponding author on reasonable request.
