# Supplementary material for: Renoprotective effect of Zhenwu decoction against renal fibrosis by regulation of oxidative damage and energy metabolism disorder
Source: Sci Rep. 2018 Oct 2;8:14627. doi: 10.1038/s41598-018-32115-9 (PMC6168532; doi:10.1038/s41598-018-32115-9)

**Renoprotective effect of Zhenwu decoction against renal fibrosis by regulation of oxidative damage and energy metabolism disorder**

Shasha Li ^a, 1^, Xue Xiao ^b, 1^, Ling Han ^a*^, Yiming Wang ^a, c^, Guoan Luo ^a, c*^

^a^ Guangdong Provincial Hospital of Chinese Medicine, No. 111 Dade Road, Guangzhou, Guangdong 510120, China.

^b^ Guangdong Metabolic Diseases Research Center of Integrated Chinese and Western Medicine, Guangdong Pharmaceutical University, Guangzhou 510006, China

^c^ Department of Chemistry, Tsinghua University, No. 30 Shuangqing Road in Haidian Distric, Beijing, 100084, China

^1^ These two authors contributed equally to the paper.

**Corresponding authors:**

Dr. Ling Han

Guangdong Provincial Hospital of Chinese Medicine, No. 111 Dade Road, Guangzhou, 510120, P.R. China.

E-mail: ertubawen@163.com; Phone/Fax: +86-20-39318678.

Prof. Guoan Luo

Guangdong Provincial Hospital of Chinese Medicine, No. 111 Dade Road, Guangzhou, 510120, P.R. China.

E-mail: [luoga@mail.tsinghua.edu.cn](mailto:luoga@mail.tsinghua.edu.cn); Phone/Fax: +86-20-39318473.

**Supplementary Figure 2. Staining results**


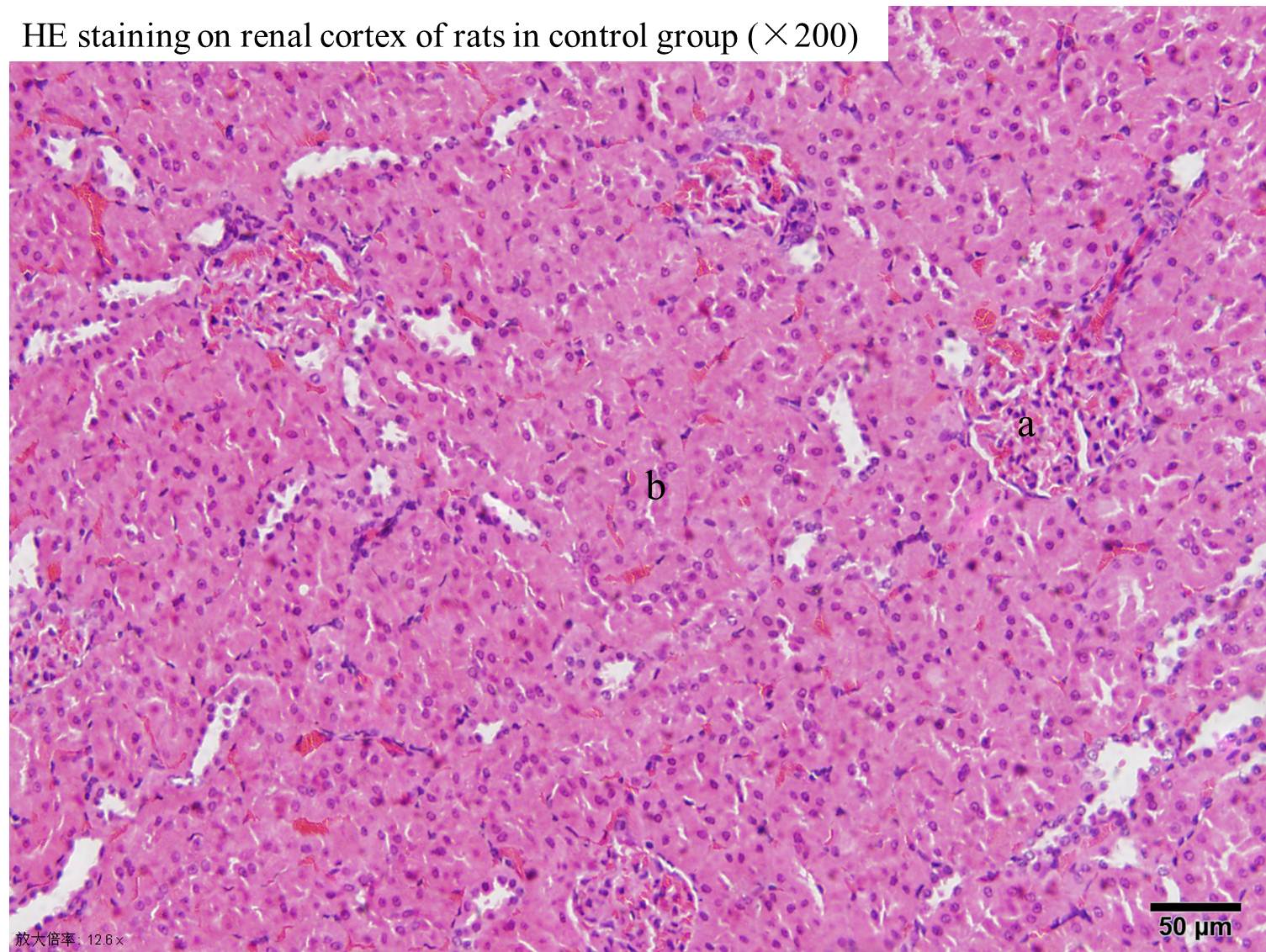


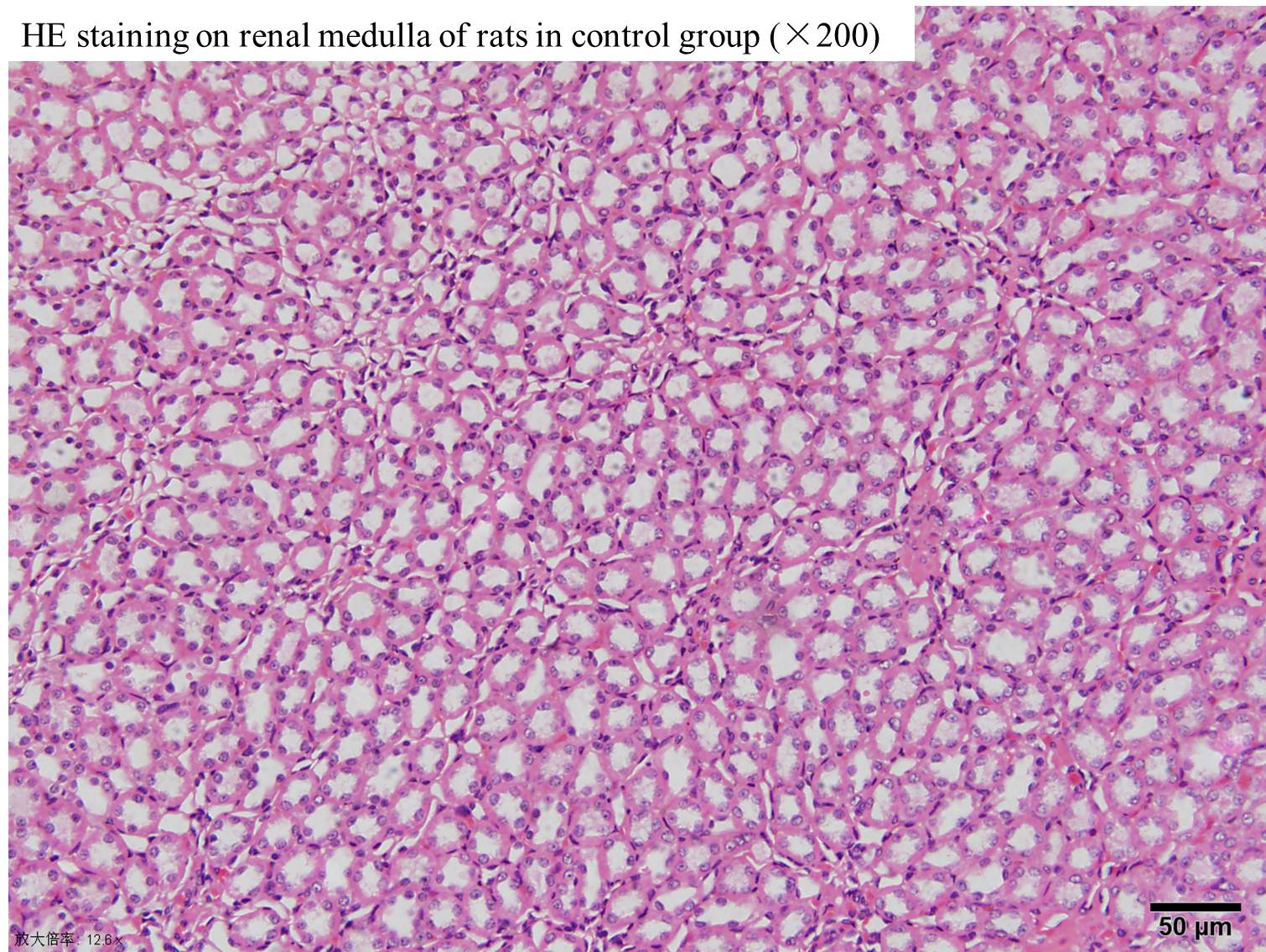


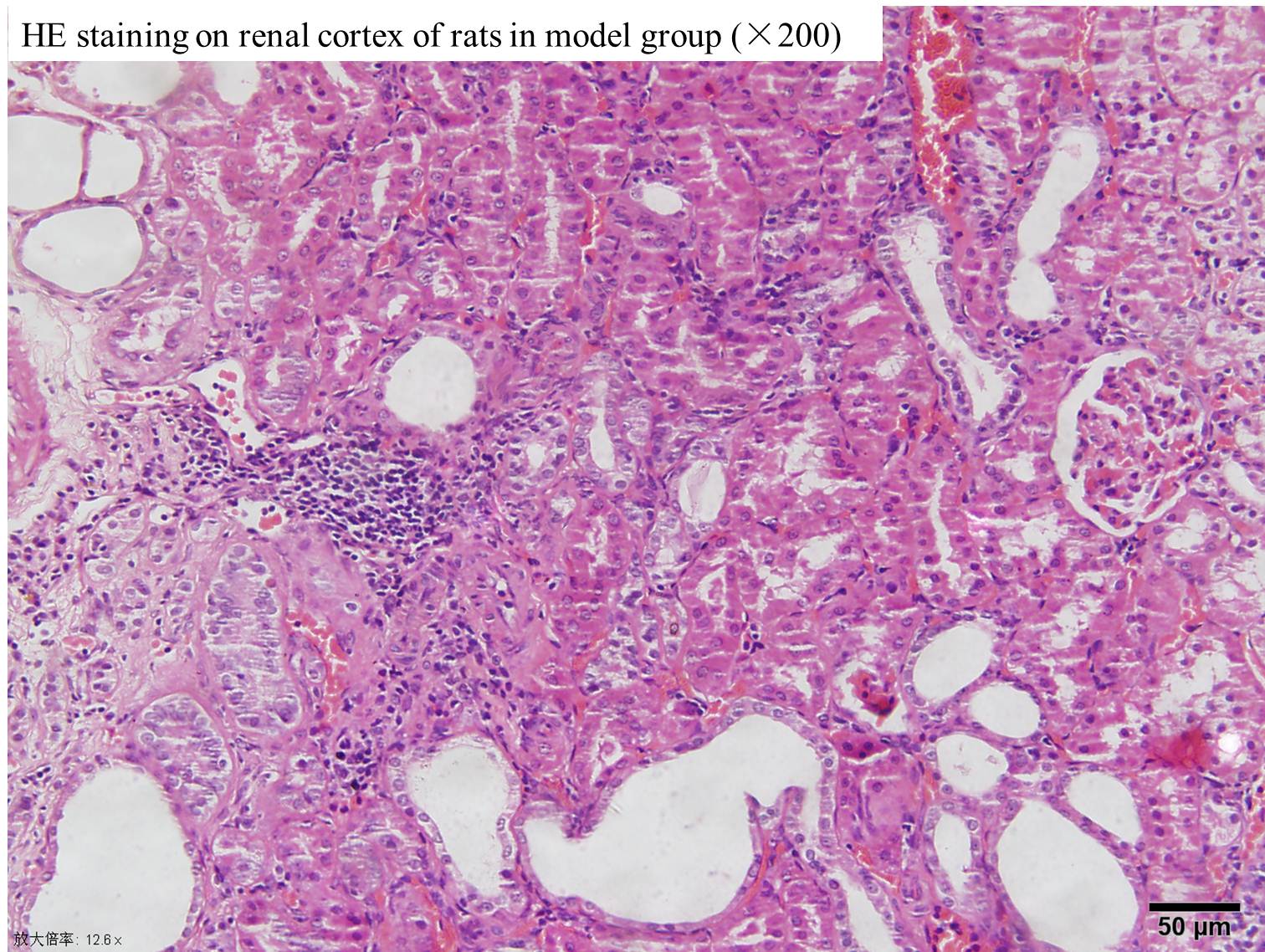


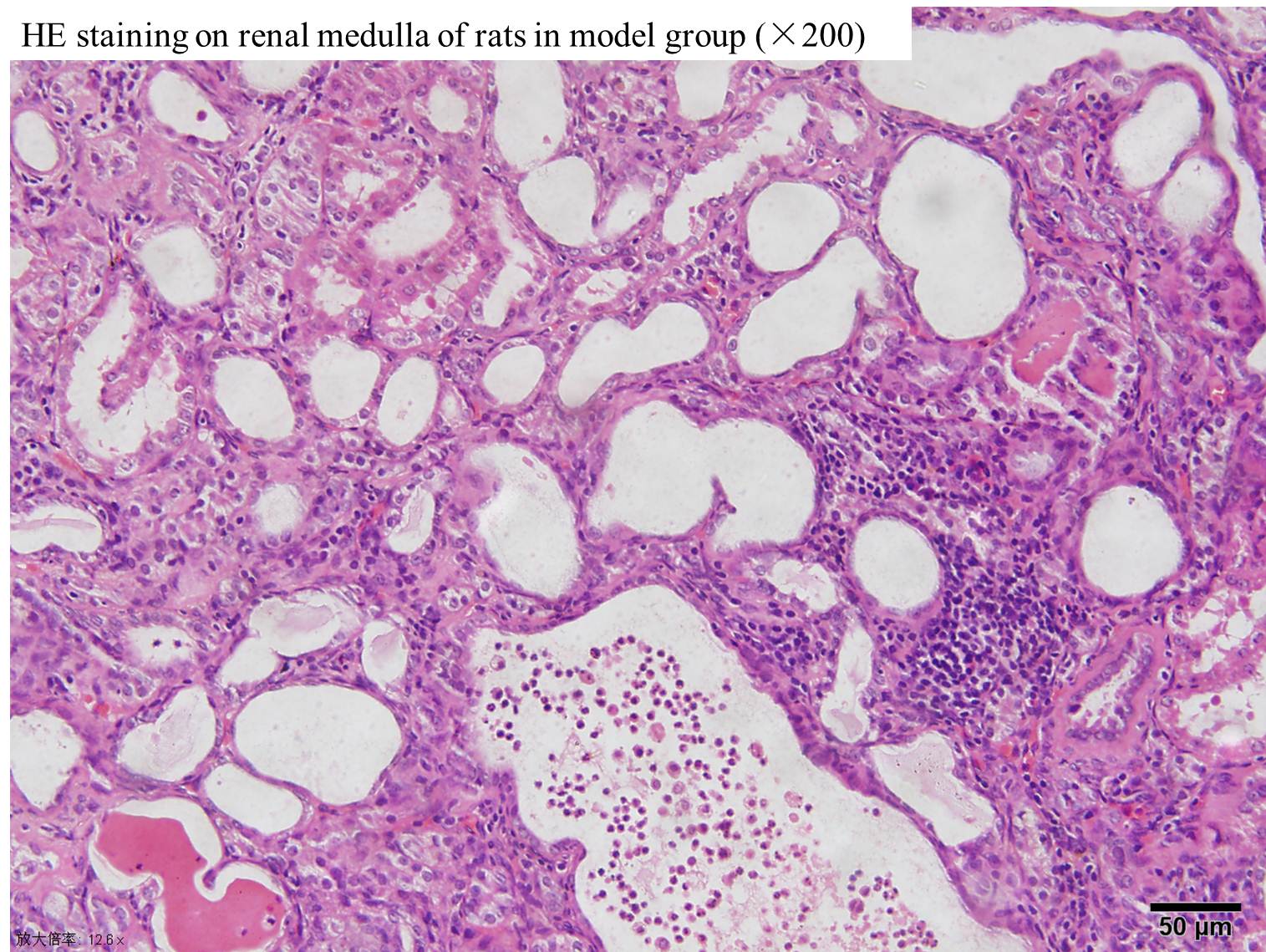


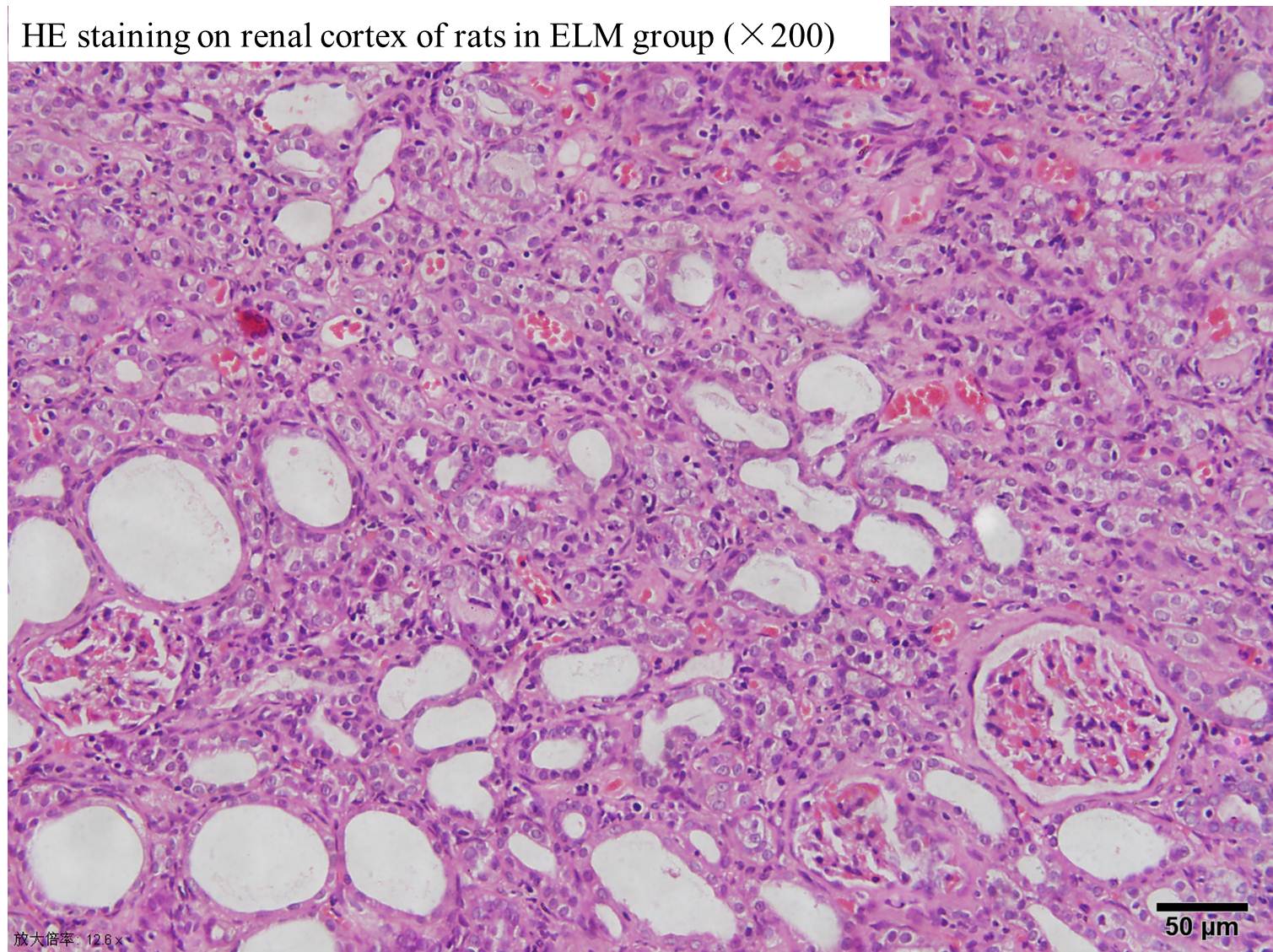


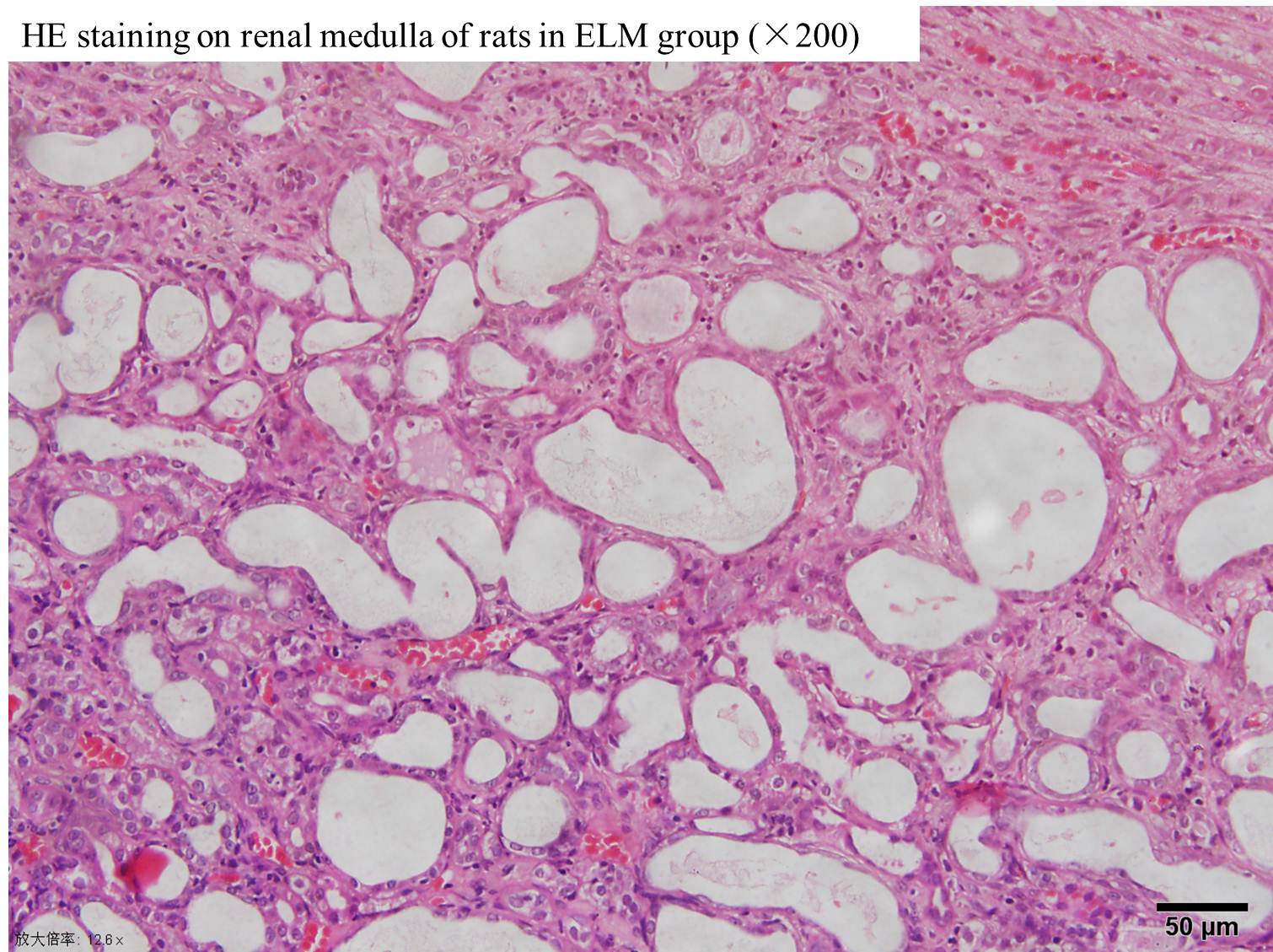


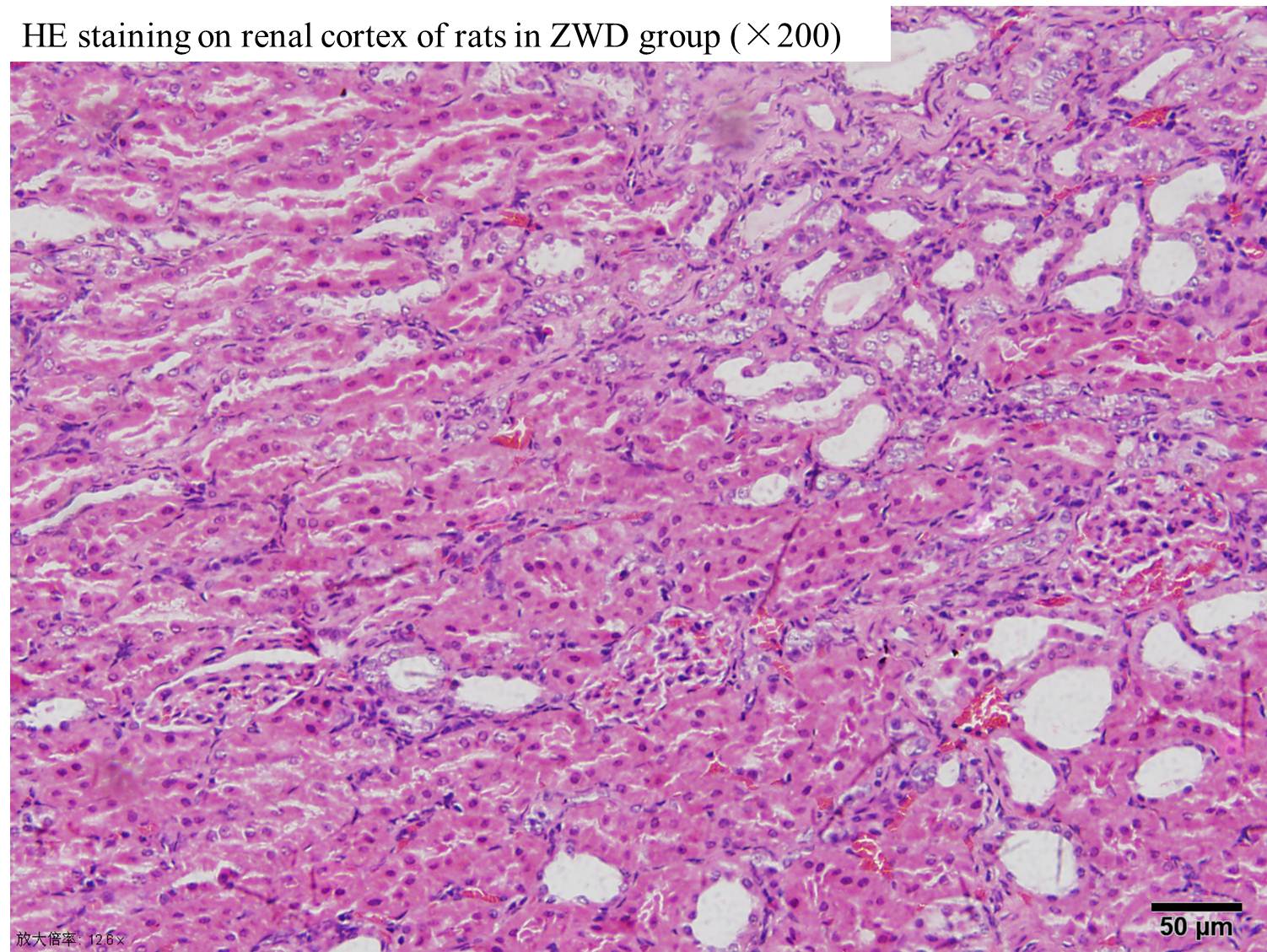


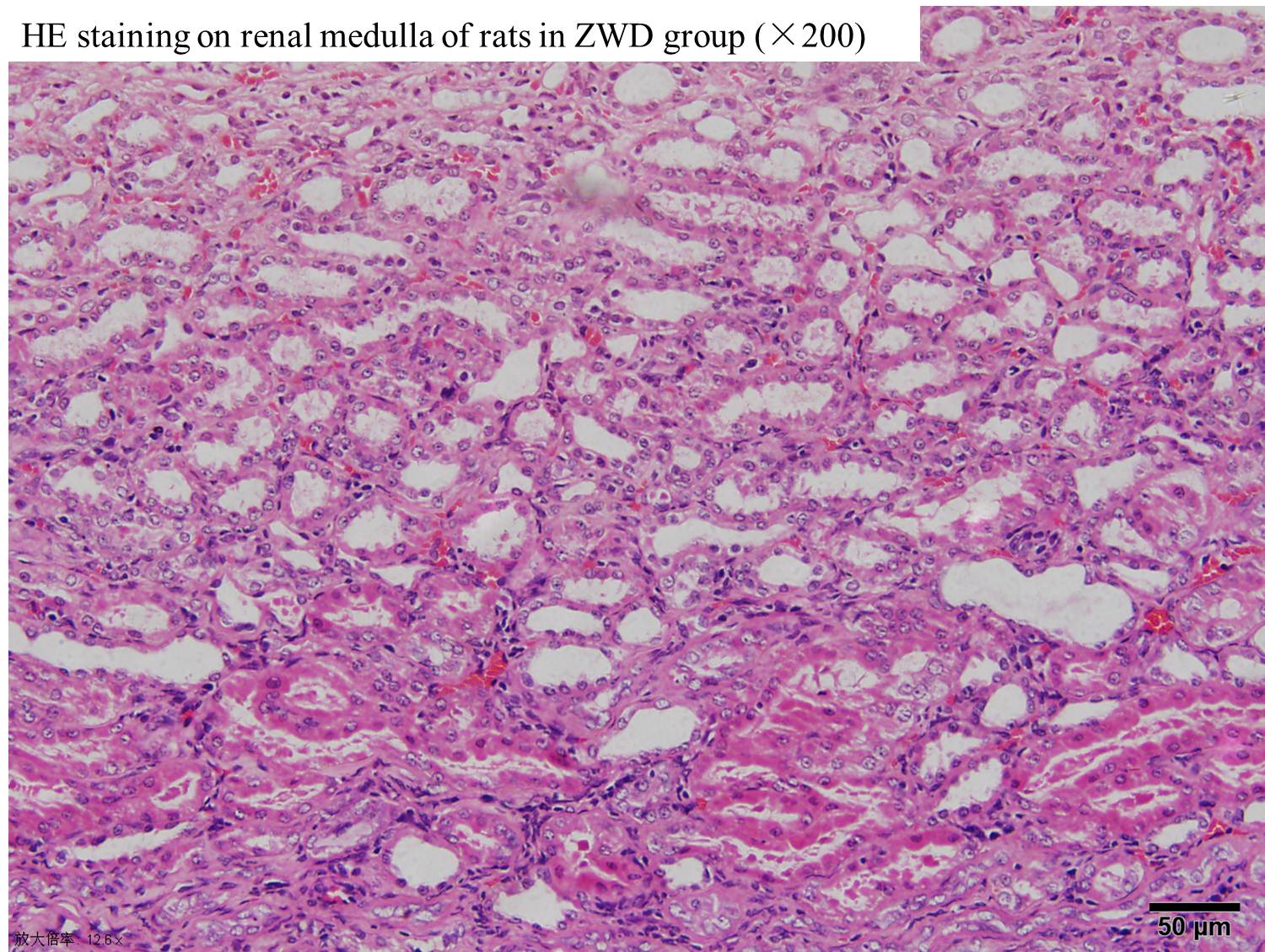


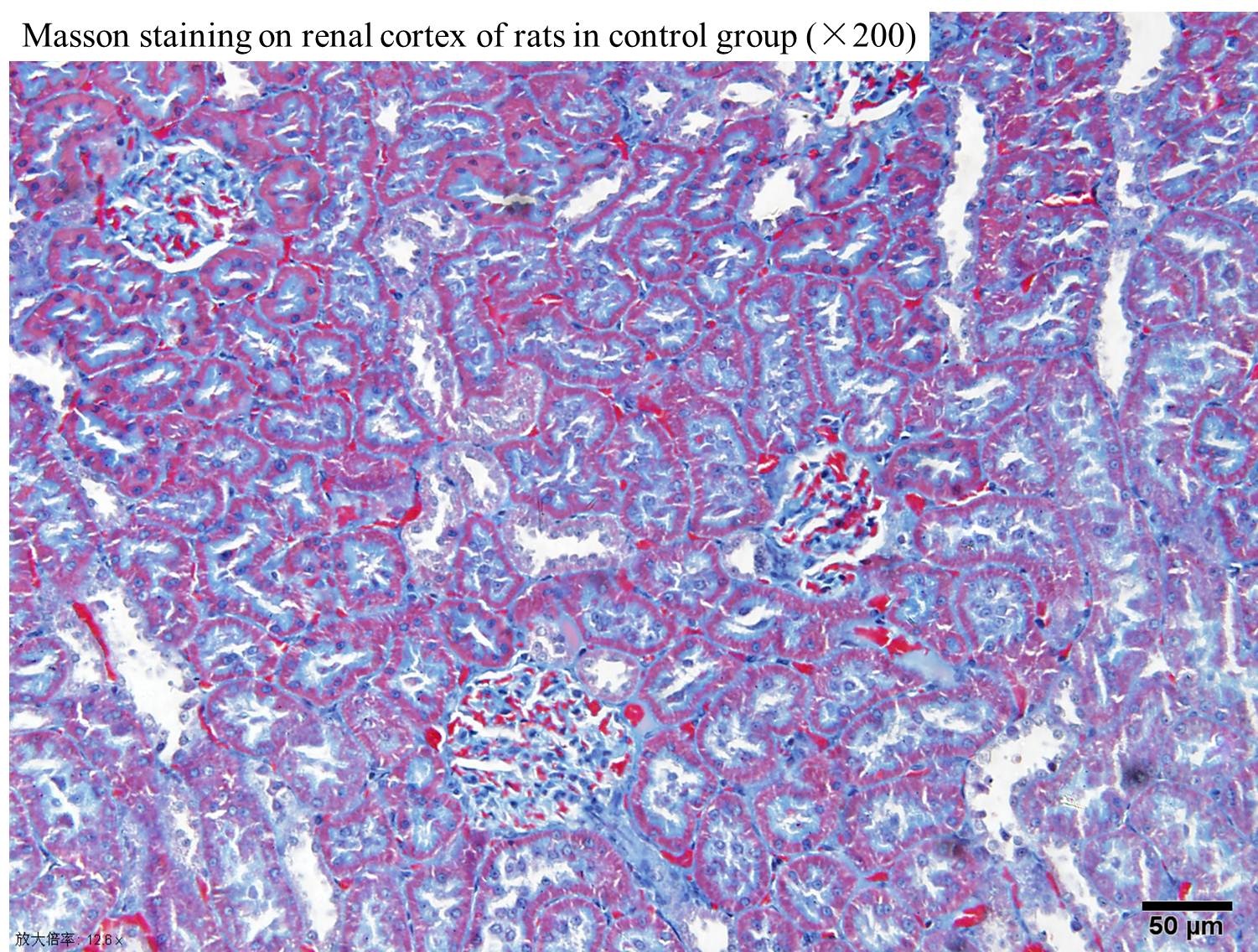


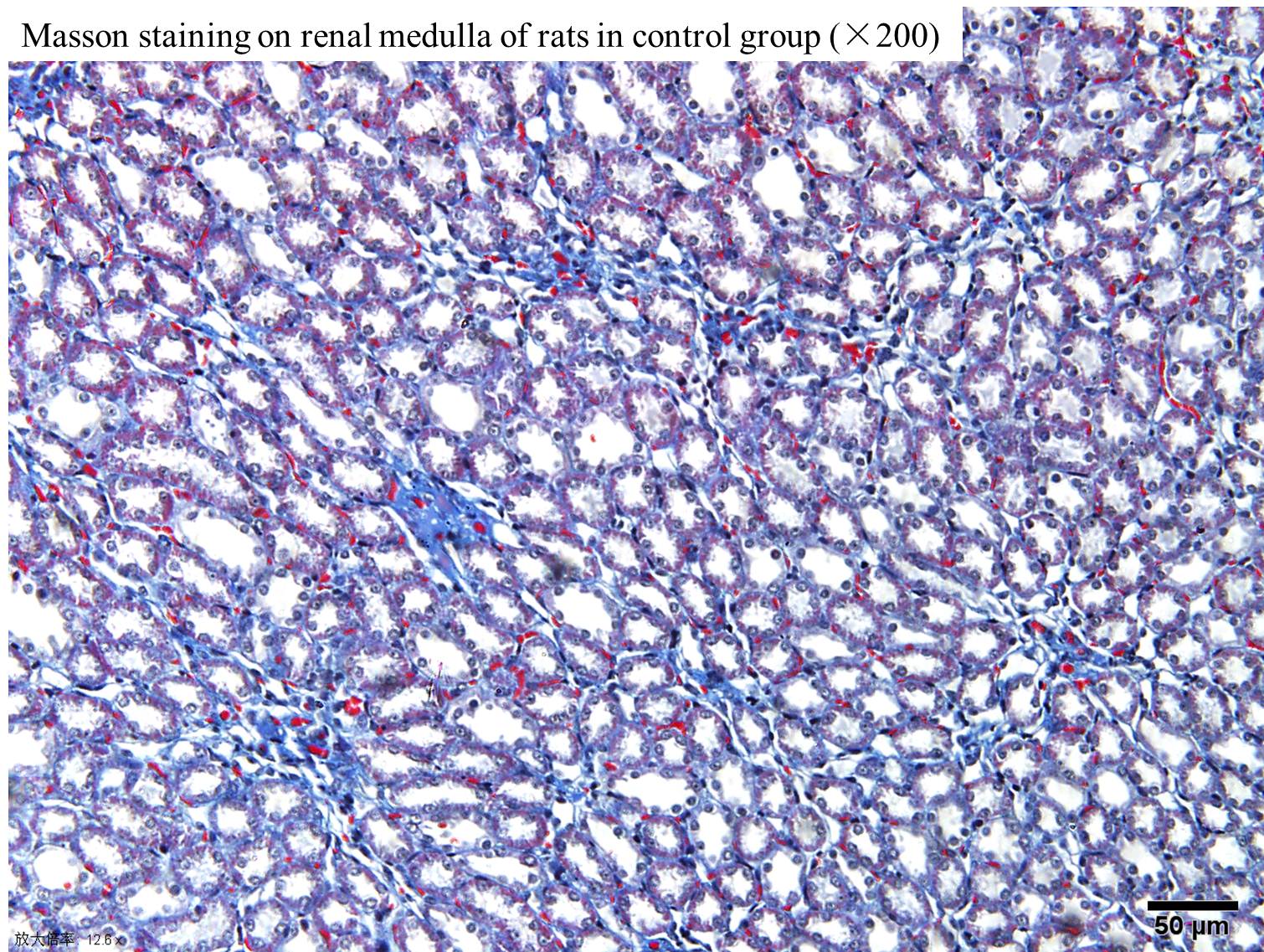


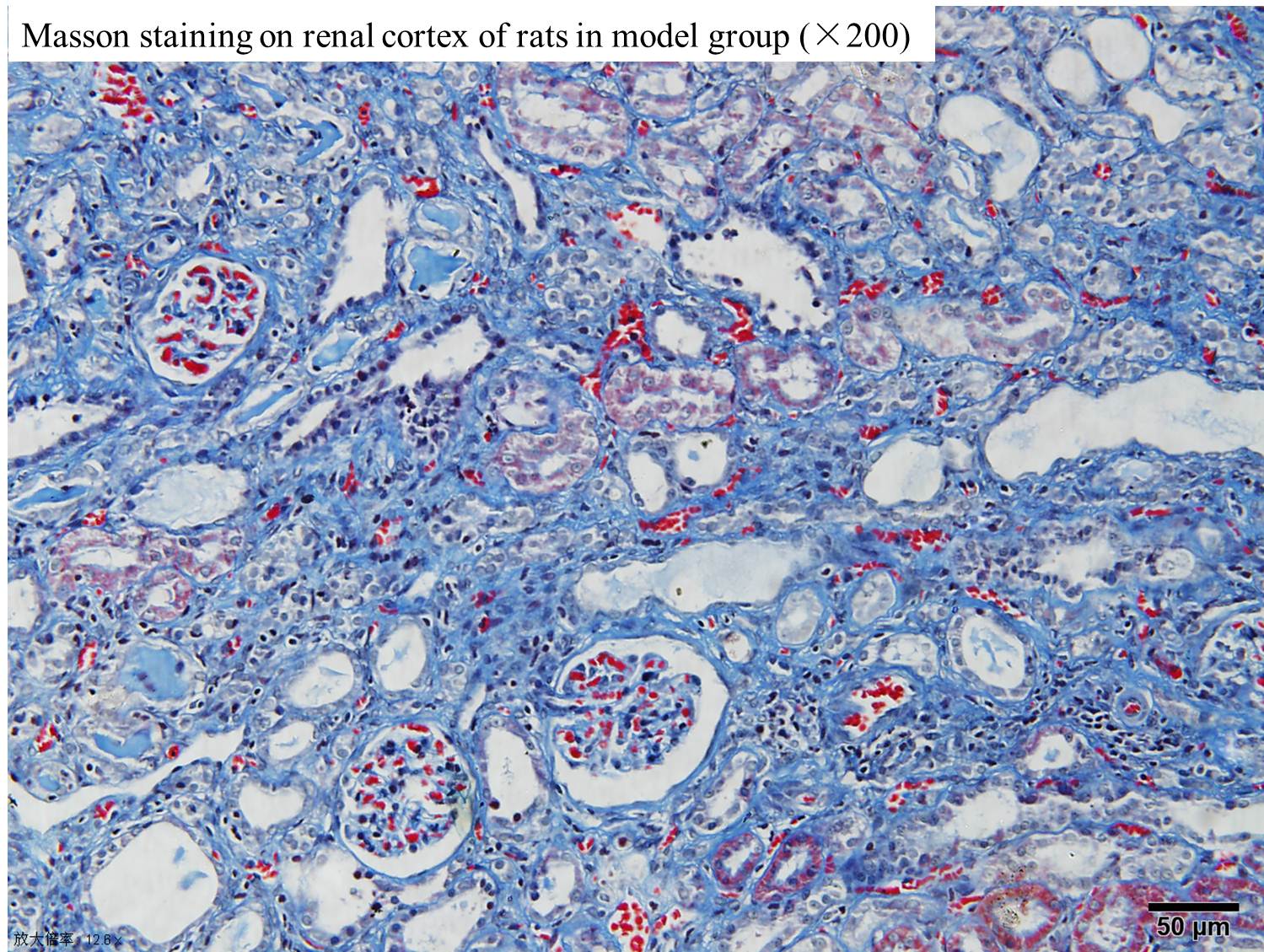


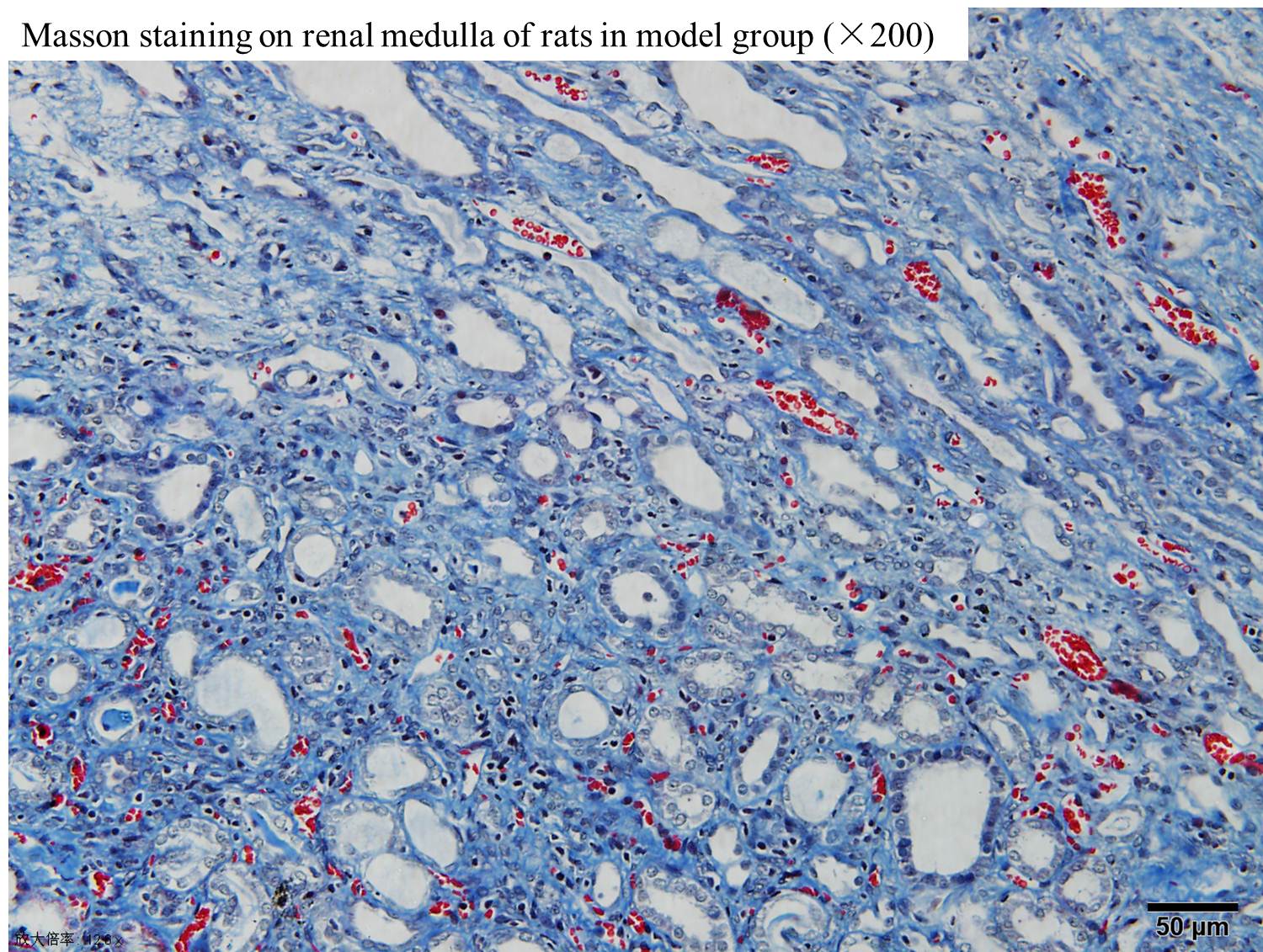


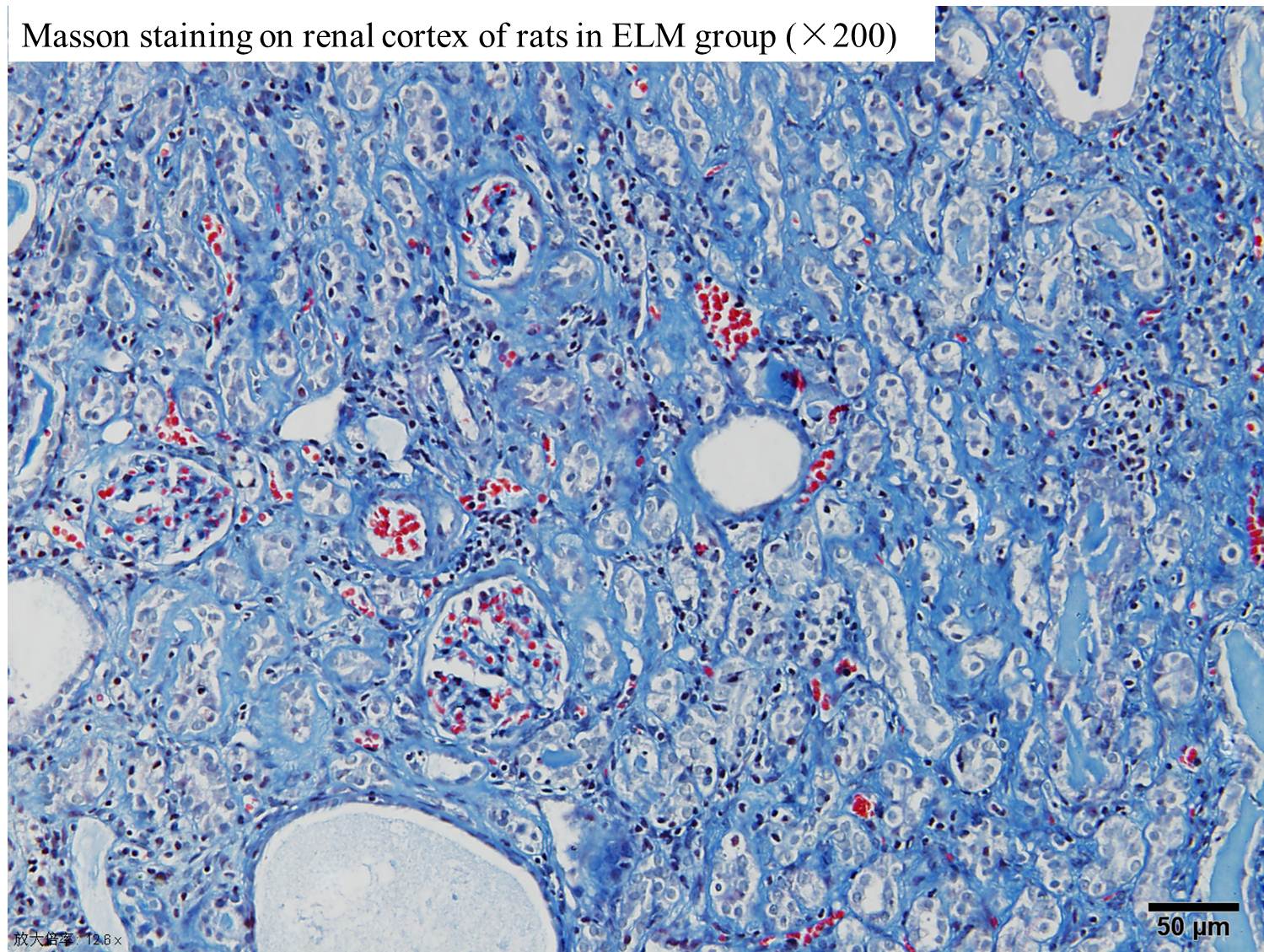


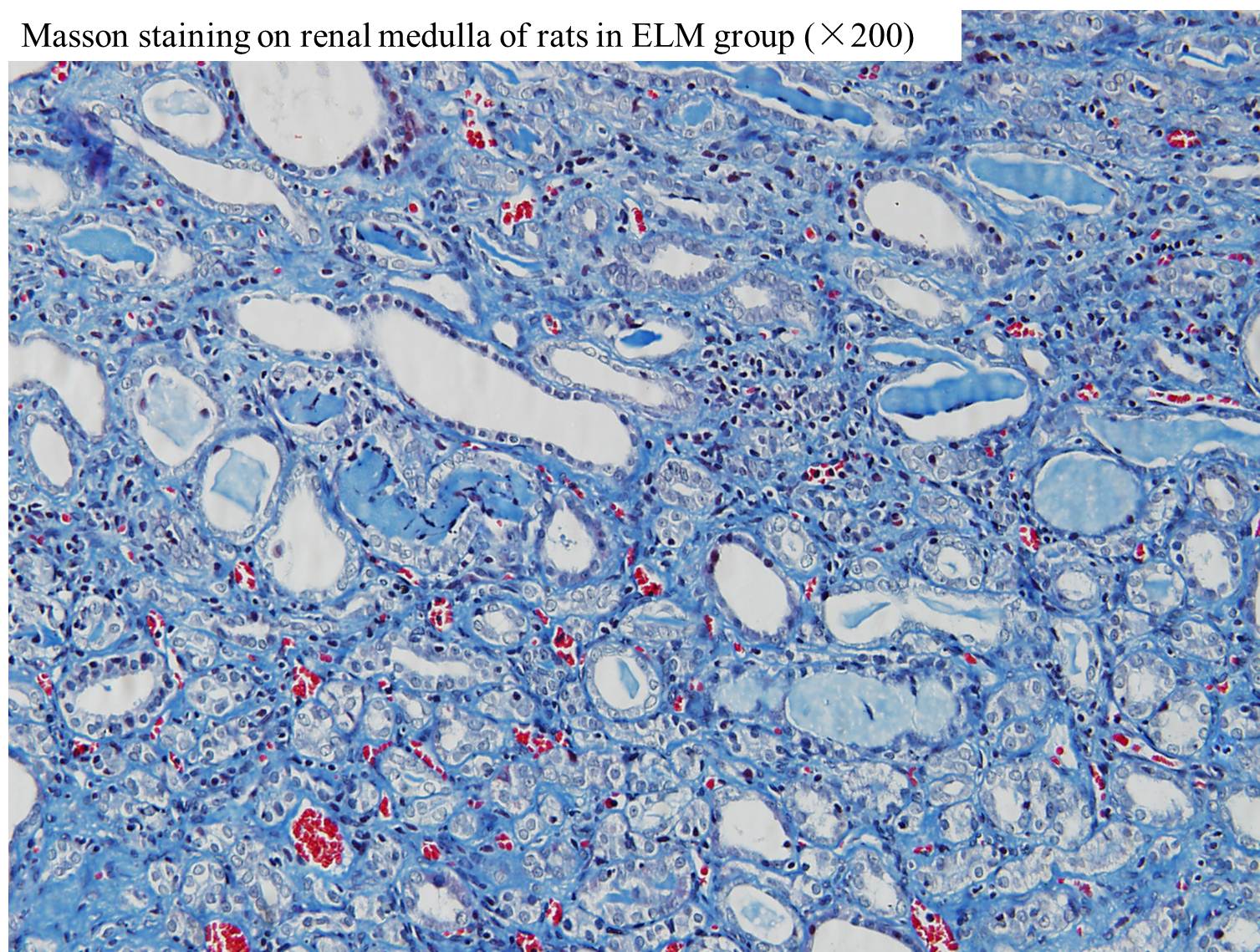


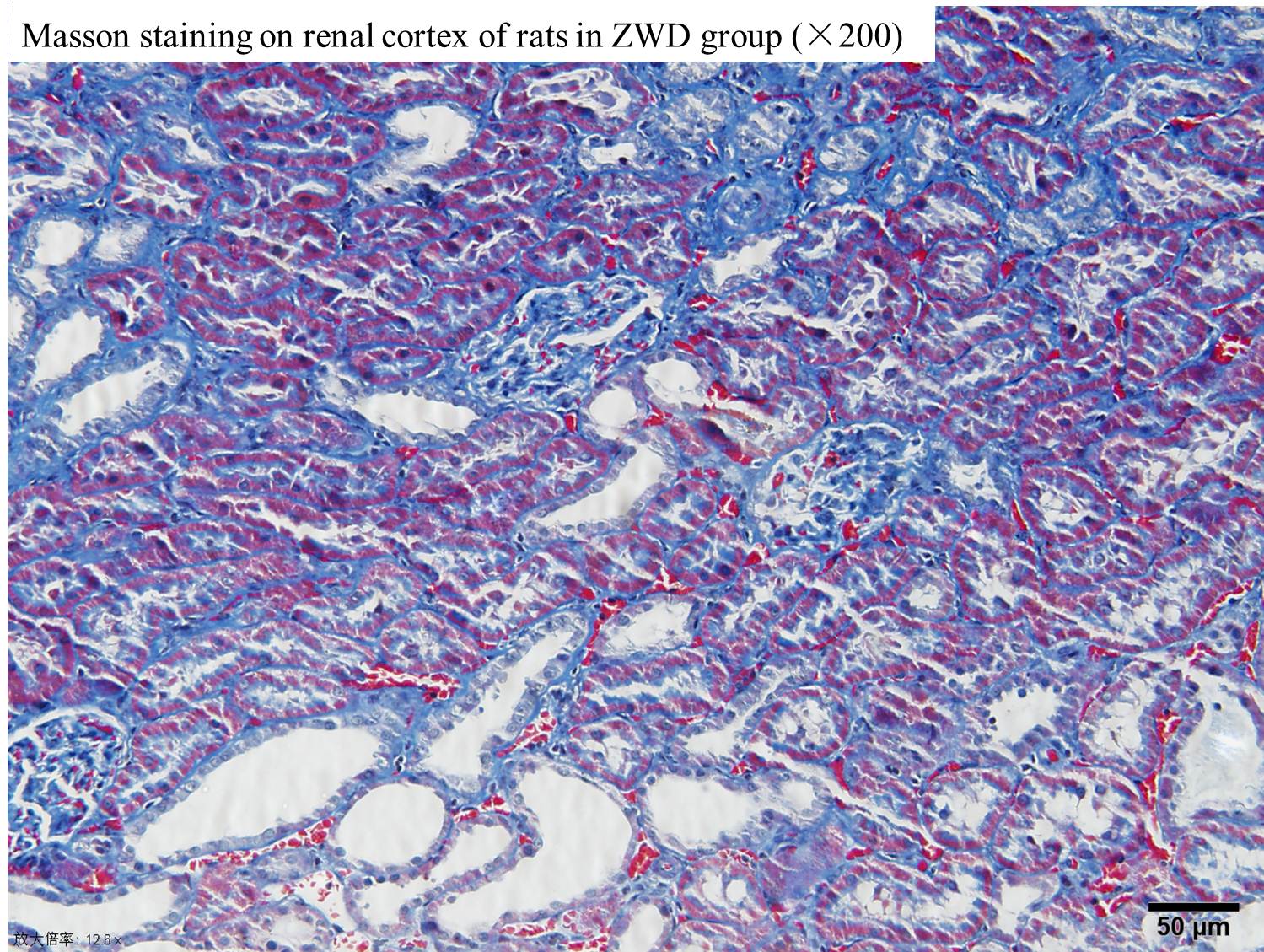


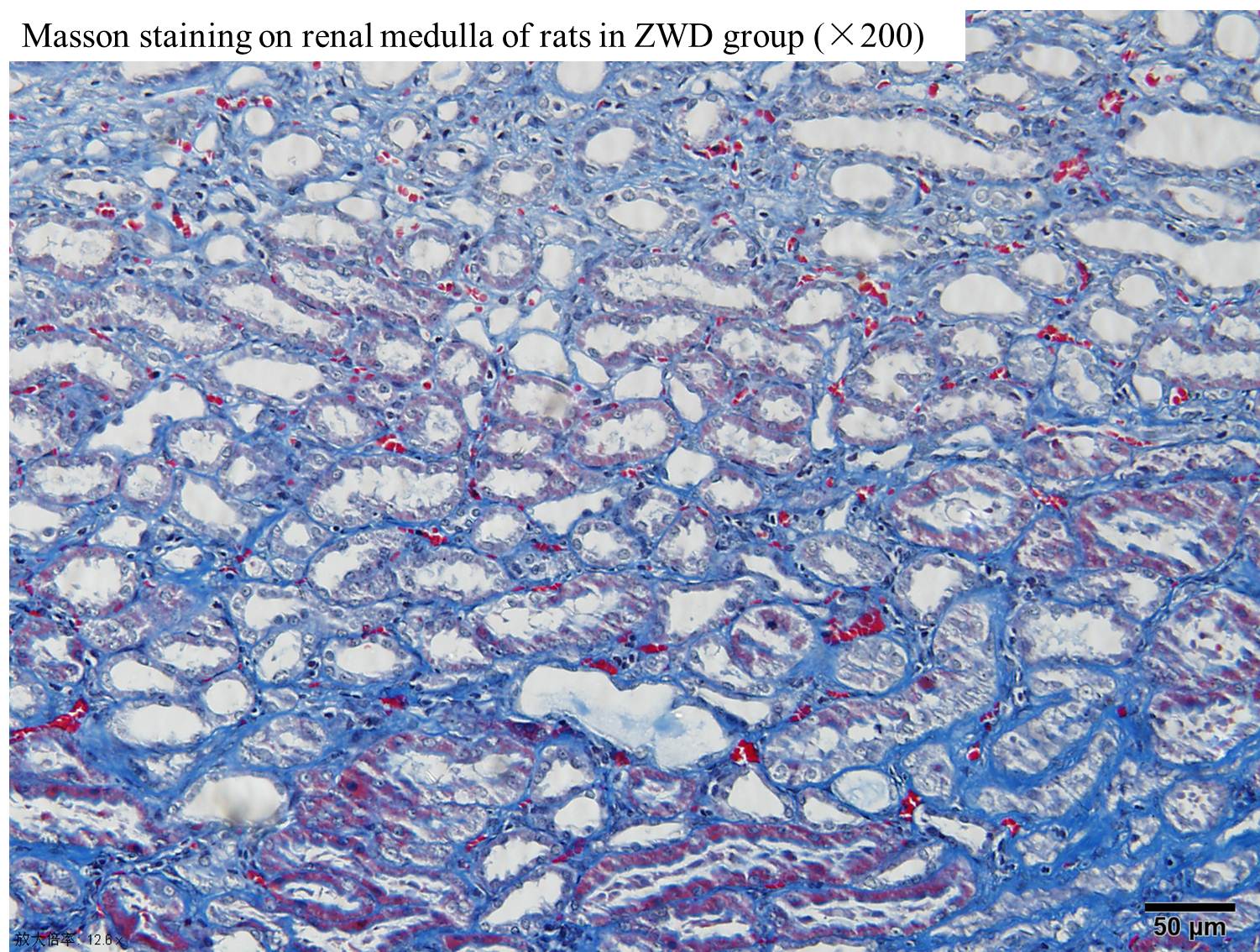

Supplement: Supplementary file 1 — Dataset 1 [file 41598_2018_32115_MOESM1_ESM.docx]
